# FAP expression as a marker of malignant transformation enabling in vivo characterization in peripheral nerve sheath tumors: a multimodal and translational study

**DOI:** 10.1007/s00401-026-02979-7

**Published:** 2026-01-27

**Authors:** Nic G. Reitsam, Alexander Gäble, Lisa Siebenhüter, Tina Schaller, Friederike Liesche-Starnecker, Eva Sipos, Sebastian Dintner, Christoph Walz, John Babic, Martin Trepel, Malte Kircher, Victoria E. Fincke, Pascal D. Johann, Bruno Märkl, Constantin Lapa, Johanna S. Enke

**Affiliations:** 1 Pathology, Faculty of Medicine, University of Augsburg, Augsburg, Germany; 2 Else Kroener Fresenius Center for Digital Health, Technical University Dresden, Dresden, Germany; 3 Bavarian Cancer Research Center (BZKF), Augsburg, Germany; 4 Nuclear Medicine, Faculty of Medicine, University of Augsburg, Augsburg, Germany; 5 Comprehensive Cancer Center, Faculty of Medicine, University of Augsburg, Augsburg, Germany; 6 Department of Neuropathology, Pathology, University of Augsburg, Augsburg, Germany; 7 Institute of Neuropathology, University Medical Center Ulm, Faculty of Medicine, Ulm University, Ulm, Germany; 8 Institute of Pathology, Faculty of Medicine, LMU Munich, Munich, Germany; 9 Ratio Therapeutics, Boston, MA USA; 10 Hematology and Oncology, Faculty of Medicine, University of Augsburg, Augsburg, Germany; 11 Pediatrics and Adolescent Medicine, Swabian Children’s Cancer Center, University Hospital Augsburg, Augsburg, Germany

**Keywords:** Peripheral nerve sheath tumors, Neurofibromatosis type 1, FAP, Fibroblast activation protein, Molecular imaging

## Abstract

Malignant peripheral nerve sheath tumors (MPNSTs) are aggressive sarcomas and a major cause of mortality in neurofibromatosis type 1 (NF-1). Distinguishing MPNSTs from benign neurofibromas remains challenging. We investigated fibroblast activation protein alpha (FAP) as a malignancy biomarker and theranostic target in peripheral nerve sheath tumors. Therefore, we integrated publicly available bulk transcriptomics, spatial transcriptomics, and single-cell RNA sequencing with immunohistochemistry (IHC) on independent archival samples. We further directly assessed clinical translatability using FAP-targeted PET/CT in an NF-1 patient undergoing work-up for suspected malignant transformation. Across independent bulk datasets, *FAP* was consistently up-regulated in MPNSTs compared with neurofibromas. In the TCGA sarcoma dataset, *FAP* varied by histotype but was clearly expressed in MPNSTs. Spatial transcriptomics revealed enrichment of *FAP*-high regions in MPNSTs and co-localization with tumor cell markers. Single-cell analysis showed *FAP* expression in MPNST tumor cells and cancer-associated fibroblasts, with the highest levels in neural crest-like tumor subpopulations previously linked to adverse prognosis; pseudotime analysis indicated decreasing *FAP* expression along trajectories toward Schwann cell precursor-like states linking *FAP* expression to a more primitive, dedifferentiated tumor cell state. IHC confirmed strong, predominantly tumor cell-intrinsic FAP expression in MPNSTs, with minimal staining in neurofibromas and normal tissues. Plexiform neurofibromas exhibited intermediate FAP expression. In clinical imaging, FAP-PET demonstrated higher tracer uptake in histologically proven MPNSTs than in benign lesions within the same patient, including a neurofibroma that was FDG-avid but FAP-negative, supporting added diagnostic specificity over FDG-PET/CT. In summary, FAP is robustly overexpressed in MPNSTs at transcript and protein levels, potentially concentrates in high-risk tumor cell states, and is detectable by targeted PET imaging. These findings identify FAP as a clinically relevant biomarker for malignancy in NF-1-associated tumors and support implementation of FAP-directed diagnostics and therapeutics in peripheral nerve sheath tumor work-up.

**Supplementary Information:**

The online version contains supplementary material available at 10.1007/s00401-026-02979-7.

## Introduction

Neurofibromatosis type 1 (NF-1) is one of the most common autosomal dominant cancer predisposition syndromes, and results from mutations in the *NF1* tumor-suppressor gene [1]. Neurofibromas, especially plexiform neurofibromas, are considered as hallmarks of NF-1, and carry significant potential of malignant transformation to a malignant peripheral nerve sheath tumor (MPNST). MPNST is an aggressive sarcoma subtype, representing the leading cause of death in NF-1 patients [2–4]. The curative treatment for MPNST is a wide marginal excision, which is only feasible if no vital structures are infiltrated. Therefore, early identification of such lesions is important.

[^18^F]-Fluorodeoxyglucose (FDG) positron emission tomography/computed tomography (PET/CT) is a well-established imaging modality in the surveillance of NF-1 patients, owing to its high sensitivity for detecting metabolically active lesions [5–10]. Together with magnetic resonance imaging (MRI), FDG-PET plays a key role in the diagnosis of malignant transformation. However, the modest specificity of radiotracer uptake can lead to false positive findings, unnecessary biopsies, and clinical uncertainty. Hence, there is a need for more selective molecular imaging methods and biomarkers that can non‑invasively distinguish malignant transformation at an early stage.

Fibroblast activation protein α (FAP) is a dipeptidyl peptidase overexpressed on cancer-associated fibroblasts (CAF) and, in several soft tissue sarcomas, directly on tumor cells [11, 12]. FAP, as an important marker of CAF, is a type II membrane-bound glycoprotein enzyme with protease activity [11, 13–16]. Preclinical studies and initial clinical reports suggest that FAP may serve both as an imaging target with FAP-directed PET tracers and as a target for radiopharmaceutical therapy or antibody-drug conjugates [17–26]. Yet, its expression (dynamics) and clinical relevance in NF-1-associated tumors remain poorly understood.

In this study, we employed a multimodal translational approach to address this gap: starting from high-throughput transcriptomic datasets, including bulk, spatial and single-cell RNA data, we profiled *FAP* gene expression across benign and malignant NF-1-associated tumors. These findings were validated by a protein-level assessment on archival tissue using immunohistochemistry (IHC). Finally, we translated these insights to the clinic by applying FAP-targeted PET/CT in an NF-1 index patient with multifocal lesions. Therefore, we provide here first translational evidence for FAP as specific non-invasive biomarker and potential theranostic target in (NF-1-associated) MPNSTs. These findings support the integration of FAP-targeted PET into NF-1 clinical studies and workflows, complementing MRI and FDG-PET in the early detection of malignant transformation.

## Methods

### Ethical approval & cohort

Our study was approved by the responsible ethical committee of Ludwig-Maximilians-University of Munich (reference: project number 25-0654) and performed in accordance with the Declaration of Helsinki. Written informed consent was obtained by the involved patients.

For analysis involving publicly available transcriptomic datasets, we refer to the original publications for details regarding ethical approval and patient consent.

Immunohistochemical analysis of FAP expression was performed on archival tissue specimens (Institute of Pathology and Molecular Diagnostics, University Hospital Augsburg). Neurofibromas (*n* = 12) exhibited typical histological features, including loosely arranged spindle cells within a variably myxoid to collagenous stroma, S100 immuno-reactivity in Schwann cells, and characteristic lattice-like CD34 staining in fibroblastic cells. In contrast, MPNSTs showed increased cellularity, nuclear atypia, and mitotic activity. Immunohistochemically, MPNSTs (*n* = 12) demonstrated reduced or focal S100 expression and were negative for other lineage-defining markers, consistent with WHO diagnostic criteria [27]. Additionally, to obtain a more comprehensive view of FAP expression across the spectrum of peripheral nerve sheath tumors, we included plexiform neurofibromas (*n* = 6; 3 with known NF-1 association, 3 without available clinical data regarding NF-1 status but with typical histopathological and immuno-histochemical marker expression) and hybrid peripheral nerve sheath tumors (*n* = 2;1 hybrid schwannoma-perineurioma, 1 hybrid neurofibroma-schwannoma) in the immunohistochemical analysis.

Given the recognized diagnostic challenges of MPNST outside an NF-1-associated setting, all cases underwent thorough re-evaluation. Available clinical data revealed NF-1 association in two cases and post-radiation etiology in one case, whereas NF-1 status could not be reliably determined in the remaining cases due to limited follow-up information. To minimize diagnostic uncertainty and exclude major fusion-driven sarcoma mimics, RNA fusion profiling was performed using the TruSight RNA Pan-Cancer Panel (Illumina, San Diego, CA, US), which did not detect sarcoma-defining fusion genes in any case. In addition, all tumors had undergone extensive immunohistochemical work-up during initial diagnostic assessment to exclude alternative lineages, using a broad marker panel adapted to the respective differential diagnoses (including, where appropriate, TLE1, desmin, FLI1, CD34, MUC4, pan-cytokeratin, melanocytic markers, GFAP, DOG1, and CD117).

Furthermore, Illumina EPIC DNA methylation profiling was performed with application of the Heidelberg sarcoma classifier [28]. In cases with sufficient DNA quantity and quality, methylation-based classification supported the diagnosis of MPNST (3 out of12 MPNSTs). In most archival samples (9 out of 12 MPNSTs), methylation profiling was not successful due to insufficient DNA yield or quality (most likely attributable to age of some specimens [> 10 years], FFPE-related degradation and small size of available biopsy material following extensive prior diagnostic work-up).

No distinction between sporadic and NF-1-associated MPNSTs was made in downstream analysis due to incomplete clinical annotation.

### Immunohistochemistry

Immunohistochemical staining for FAP was performed on a Ventana BenchMark ULTRA platform using the iVIEW DAB detection system (Roche, Mannheim, Germany). A recombinant anti-FAP antibody (clone ab207178, Abcam, Cambridge, UK) was applied at a dilution of 1:250. Antigen retrieval was carried out with CC1 buffer for 92 min, followed by primary antibody incubation at 37 °C for 32 min. Detection was performed according to the manufacturer’s protocol.

We used the H-score for quantification of FAP immunohistochemistry: 1 × percentage of weak staining) + (2 × percentage of moderate staining) + (3 × percentage of strong staining). The H score ranges from 0 to 300 [29]. IHC stains were evaluated together by two pathologists (BM, NGR).

### Bulk, spatial, and single-cell transcriptomic data analyses

**Fig. 1:**
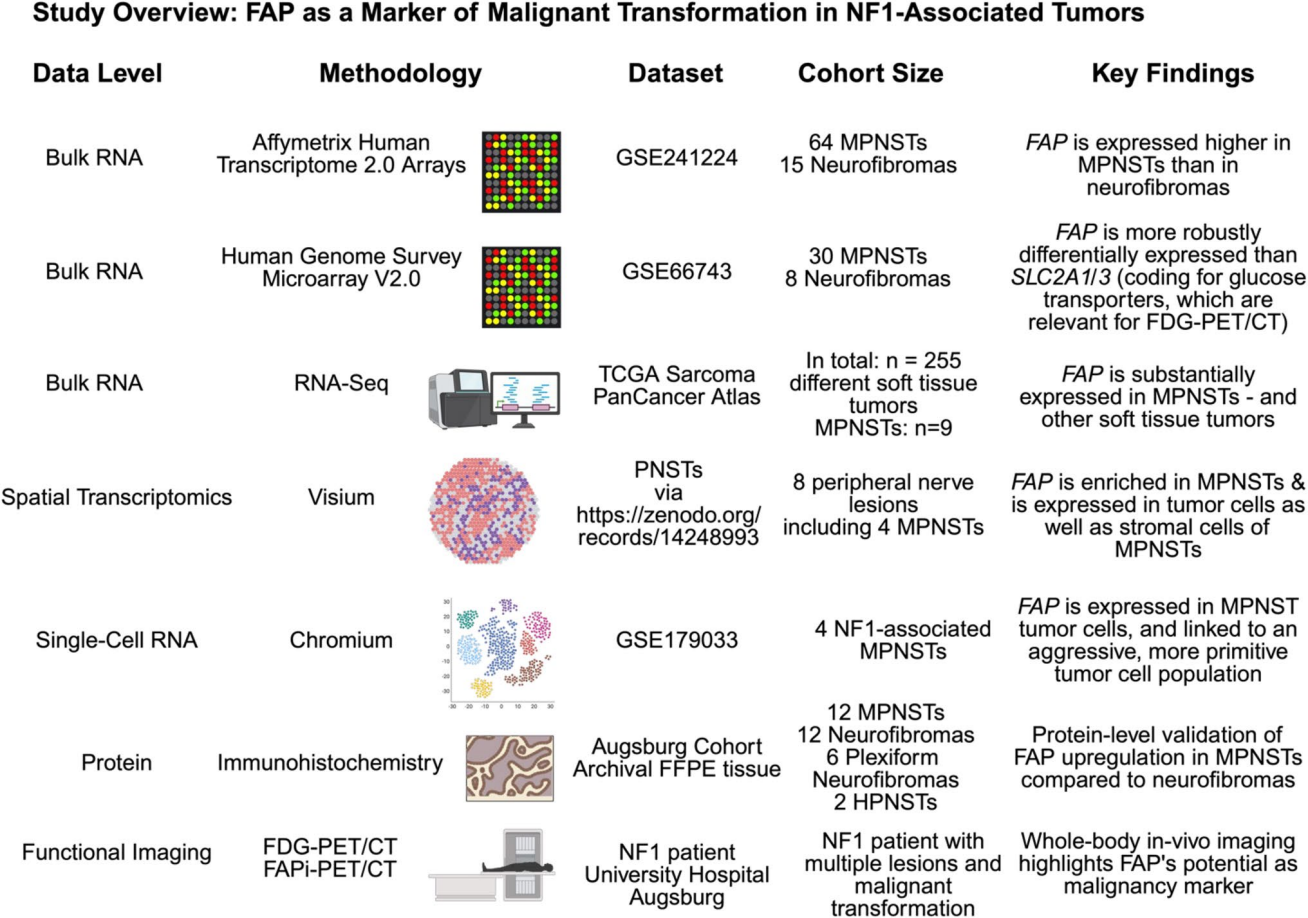
Study overview. To prove FAP as potent biomarker for distinguishing MPNSTs from neurofibromas and hence to provide a rationale for FAP-directed PET/CT in the diagnostic work-up of NF-1 patients, we investigated five publicly available datasets spanning different modalities (bulk RNA profiling by array- and RNA-seq-based technologies, spot-based spatial transcriptomics and single-cell RNA profiling). We validated these RNA-level findings on protein level by immunohistochemistry. Finally, we provided clinical validation of FAP as a malignancy biomarker using FAP-directed PET/CT in an NF-1 patient. Created in BioRender. Reitsam, N. (2026) https://BioRender.com/wvtr7fz

We performed a multimodal analysis of FAP expression in NF-1-associated lesions. The study outline is summarized in Fig. 1.

To investigate the differences in *FAP* gene expression between neurofibromas and MPNSTs, publicly available gene expression datasets *(GSE241224, GSE66743)* [30, 31] were retrieved from the Gene Expression Omnibus (GEO). Expression matrices and corresponding phenotype metadata were processed using the GEOquery and Biobase R packages. *FAP, SLC2A1, SLC2A3* mRNA expression was extracted based on the following probe IDs: *FAP*: TC02002480.hg.1, *SLC2A1*: TC01002578.hg.1, *SLC2A3*: TC12001170.hg.1; *FAP*: hCG1685968.2; *SLC2A1*: hCG40024.3; *SLC2A3*: hCG22218.2 and hCG1730826.1). Samples were annotated as neurofibroma or MPNST based on sample metadata. Gene-wise expression comparisons between tumor types were performed using unpaired two-tailed t tests. Additionally, *FAP* gene expression across sarcoma subtypes in the TCGA dataset (*n* = 255) [32–34] was analyzed using batch-normalized RSEM RNA-seq data; differences were assessed by Kruskal–Wallis test followed by Dunn’s post hoc test with Bonferroni correction for multiple comparisons.

We analyzed spatial transcriptomics data from eight peripheral nerve lesions profiled by the 10× Genomics Visium platform, originally published by *Bremer *et al*.* [35]. These included four malignant peripheral nerve sheath tumors (MPNSTs), two hybrid peripheral nerve sheath tumors (HPNSTs), one plexiform neurofibroma, and one NF2-associated neuropathy. We processed the data using Seurat [36, 37] (Version: 5.3.0, https://satijalab.org/seurat/) in R (Version: 4.4.0). For each sample, we retained spatial spots with > 50 detected genes and < 20,000 unique molecular identifiers (UMIs). Data were log-normalized and 2,000 highly variable genes were identified using the *vst* method. Each sample was annotated with lesion type metadata and subsequently merged into a single Seurat object for downstream analysis. To identify regions with elevated *FAP* expression, we classified spatial spots as “high FAP” if expression exceeded the 75th percentile across all spots. Enrichment of high FAP expression in MPNST versus non-MPNST samples was tested using a 2 × 2 contingency table and Fisher’s exact test. To explore the biological context of *FAP* expression, we performed a focused co-expression analysis across all MPNST samples. We tested co-enrichment of *FAP* with MPNST markers (*NGFR, APOD, L1CAM, S100B, CDK4, VIM, ZEB1, MYH9, SOX10*) and stromal markers (*PDGFRB, DCN, COL1A1, LAMA2*). For each marker, we binarized expression per spot (nonzero vs zero). We calculated the percentage of high-FAP spots that were also positive for each marker (“% Joint + ”) to approximate spatial co-localization. This statistical framework emphasizes the spatial relationship between *FAP* and known tumor biology markers in MPNST.

Single‐cell RNA‐sequencing (scRNA-seq) data from four NF-1‐associated MPNST samples [38] were processed in R (Version: 4.4.0) using Seurat (Version: 5.3.0). Raw UMI count matrices were loaded via *Read10X*, filtered to remove cells with < 200 or > 9,000 genes and > 20% mitochondrial reads, and normalized with the *LogNormalize* method; mitochondrial percentage was regressed out during scaling. Highly variable features (n = 2,000) were identified using the *vst* method, followed by PCA (30 PCs) and UMAP (dims 1:20) for dimensionality reduction. Shared‐nearest‐neighbor graphs were constructed (*FindNeighbors*, dims 1:20) and clusters were defined with a resolution of 0.1 (*FindClusters*). Differential expression analysis (*FindAllMarkers*, min.pct = 0.25, log2FC > 0.25) yielded the top 10 marker genes per cluster, which were then manually annotated to distinguish tumor (e.g., mesenchymal‐like, cycling Schwann‐lineage) from immune, stromal, and vascular populations. Cell‐type compositions and marker expression *(FAP, SLC2A1, SLC2A3)* were visualized by UMAP and DotPlot. To explore differentiation trajectories among tumor clusters, we applied Slingshot [39] (Version: 2.12.0) (slingshot.html) on UMAP embeddings.

Bulk, spatial, and single-cell transcriptomic analyses were performed using custom R scripts, which are available on GitHub (https://github.com/ngr-path/Analysis_R_FAP_NF1_MPNST).

### Statistical analysis

Continuous data are given as mean ± standard deviation or median and range, where appropriate. Comparisons were performed using unpaired two-tailed t tests for bulk RNA data and Wilcoxon rank-sum tests for immunohistochemical H-scores. Fisher’s exact tests were used for spatial enrichment analyses, and co-expression and trajectory inference in spatial and single-cell transcriptomics were performed using Seurat and Slingshot in R as outlined in the corresponding subsections. Bonferroni correction and Benjamini-Hochberg procedure were performed to adjust for multiple testing. A p value of < 0.05 was considered as statistically significant.

### PET/CT imaging

Scans were obtained on a dedicated PET/CT scanner (Biograph mCT 40; Siemens Healthineers, Erlangen, Germany). After administration of 179 MBq [^68^ Ga]Ga-LNTH-1363S, (FAP-directed radiotracer; mass dose, 90 ug) or 167 MBq of [^18^F]FDG, respectively, whole-body imaging was acquired at 60 min post administration with 6–8 bed positions and an emission time of 2 min each. Data were decay-corrected to the starting time of each individual scan. PET imaging was accompanied by whole-body auxiliary CT (35 mAs, 120 keV, a 512 × 512 matrix, 5-mm slice thickness, increment of 30 mm/s, rotation time of 0.5 s, and pitch of 0.8) for attenuation correction purposes. Contrast-enhanced CT of the upper and lower body was acquired with FDG-PET imaging. Images were reconstructed using a standard reconstruction protocol provided by the manufacturer (2 iterations, 21 subsets) and corrected for randoms, scatter, decay, and attenuation.

## Results

### FAP is upregulated in MPNSTs compared to neurofibromas and represents a potential therapeutic target expressed by tumor cells

To comprehensively assess *FAP* gene expression in peripheral nerve sheath tumors, we systematically interrogated publicly available transcriptomic datasets spanning multiple platforms including array-based and RNA-seq-based bulk gene expression as well as spatial and single-cell transcriptomics.

We re-analyzed *FAP* gene expression across several soft tissue tumor subtypes in the TCGA sarcoma dataset [32] (*n* = 255; batch-normalized RNA-seq data generated with RSEM [40]). There was a differential *FAP* gene expression between different soft tissue tumor types (Kruskal-Wallis test, *p* < 0.001, Supplementary Figure S1) with a relevant *FAP* expression in MPNSTs (*n* = 9). Synovial sarcomas (*n* = 10) exhibited the lowest *FAP* expression, while desmoid tumors showed a very high *FAP* expression, indicating that there is a relevant heterogeneity of *FAP* expression across different soft tissue tumor subtypes. Given the small sample size of included desmoid tumors (*n* = 2), this observation should be interpreted with caution; however, it aligns with prior reports of FAP protein overexpression in desmoid tumors [41]. Results of pairwise comparisons using Dunn’s test with Bonferroni correction are summarized in Supplementary Table S1.

**Fig. 2:**
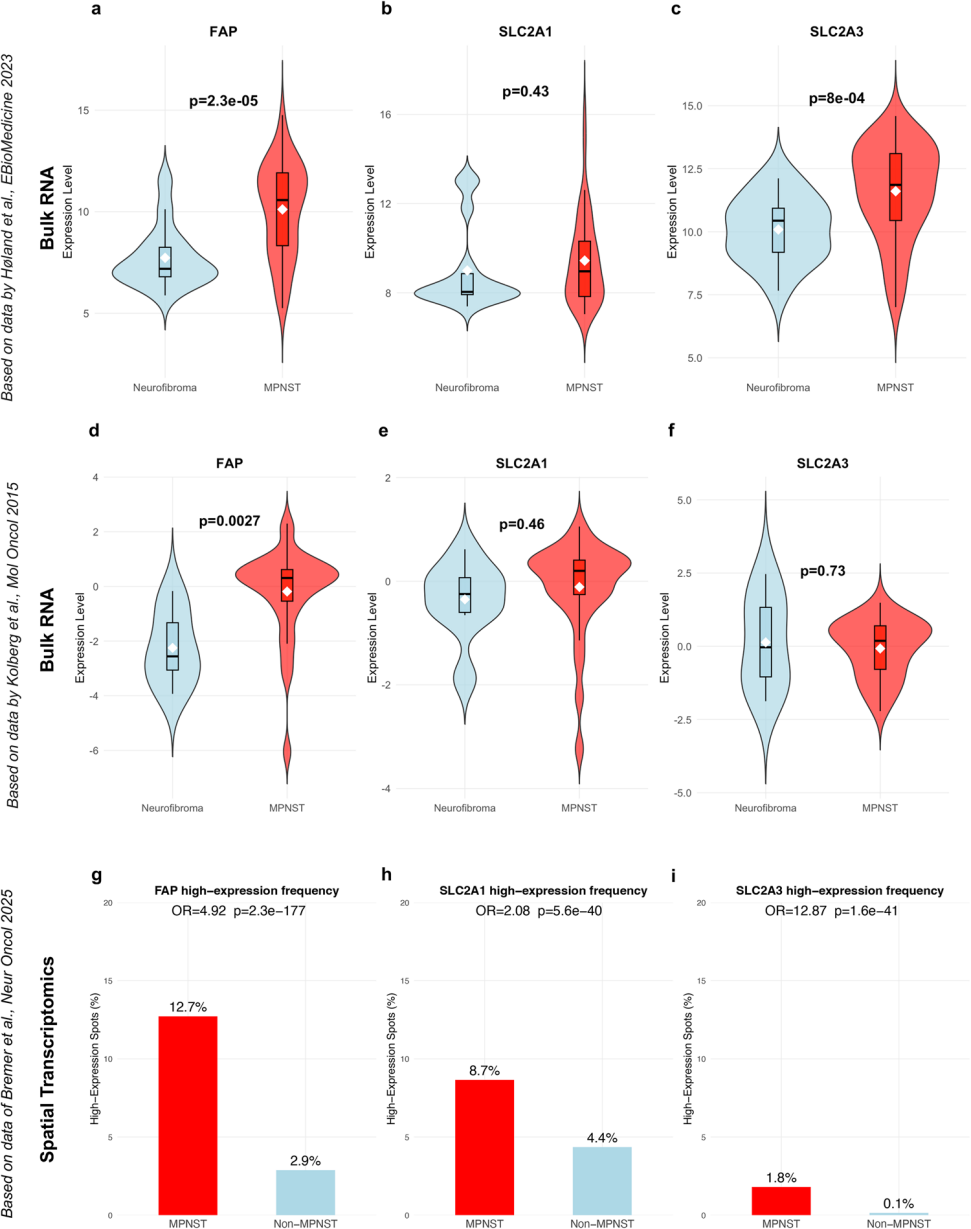
Gene expression profiling proves *FAP* upregulation in MPNSTs compared to neurofibromas on RNA level. a-f Bulk gene expression profiling across two different cohorts (*GSE241224*: MPNST, *n* = 64, neurofibroma, *n* = 15; *GSE66743*: MPNST, *n* = 30, neurofibroma, *n* = 8). *FAP* gene expression is significantly higher in MPNST than in neurofibromas in both datasets, whereas there is no difference for *SLC2A1* gene expression (encoding for GLUT1). *SLC2A3* (encoding for GLUT3) is higher expressed in MPNST in one dataset. g-i Bar plots showing the proportion of spatial transcriptomic spots with high expression (top 25%) of *FAP*, *SLC2A1*, and *SLC2A3* across MPNST and non-MPNST (benign nerve lesions) samples. High-expression status was defined using the 75th percentile threshold per gene. Odds ratios (OR) and p values were derived from Fisher’s exact test comparing frequency of high-expression spots between lesion types

Array-based bulk gene expression profiling (*GSE241224*) [30] reveals significant upregulation of *FAP* gene expression in MPNSTs (n = 64) compared to neurofibromas (*n* = 15; *p* < 0.001, Fig. 2a). Consistent with prior FDG-PET/CT studies demonstrating elevated glucose uptake in MPNSTs [5–7, 9, 10], we observed increased *SLC2A3* (coding for GLUT3) expression (*p* < 0.001, Fig. 2c), while *SLC2A1* (coding for GLUT1) was not significantly different compared to neurofibromas (*p* = 0.43, Fig. 2b). We aimed to validate these findings in another independent cohort profiled by array-based gene expression analysis (*GSE66743* [31], Fig. 2 d-f) and could observe an increased *FAP* expression in MPNSTs (*n* = 30) compared to neurofibromas (*n* = 8) in this smaller dataset (*p* = 0.0027). There was no difference between MPNST and neurofibroma samples in *SLC2A1* and *SLC2A3* gene expression (*p* = 0.46 and *p* = 0.73).

To further contextualize our gene expression findings, we refer here to previously published transcriptomic data comparing GTEx normal peripheral nerve tissue with TCGA MPNSTs (see in their manuscript [42] in *Supplementary Table S12*). Although this comparison involved normal nerve rather than neurofibroma, *FAP* was significantly up-regulated in MPNSTs (log₂ fold-change = 2.66; adjusted *p* < 0.001) [42], supporting its consistent overexpression in malignant peripheral nerve sheath tumors relative to non-malignant peripheral nerve-derived tissues.

We note that in a limited dataset reported by *Miller *et al*.* [43] (MPNST *n* = 6; plexiform neurofibroma *n* = 13), *FAP* expression appeared heterogeneous and in some cases at similar levels in plexiform neurofibromas and MPNSTs (Supplementary Figure S2). While very limited by sample size, this is biologically plausible since plexiform neurofibromas carry the highest risk of malignant transformation [44] and support the concept that elevated FAP expression may be linked not only to malignancy but also to progression risk.

**Fig. 3:**
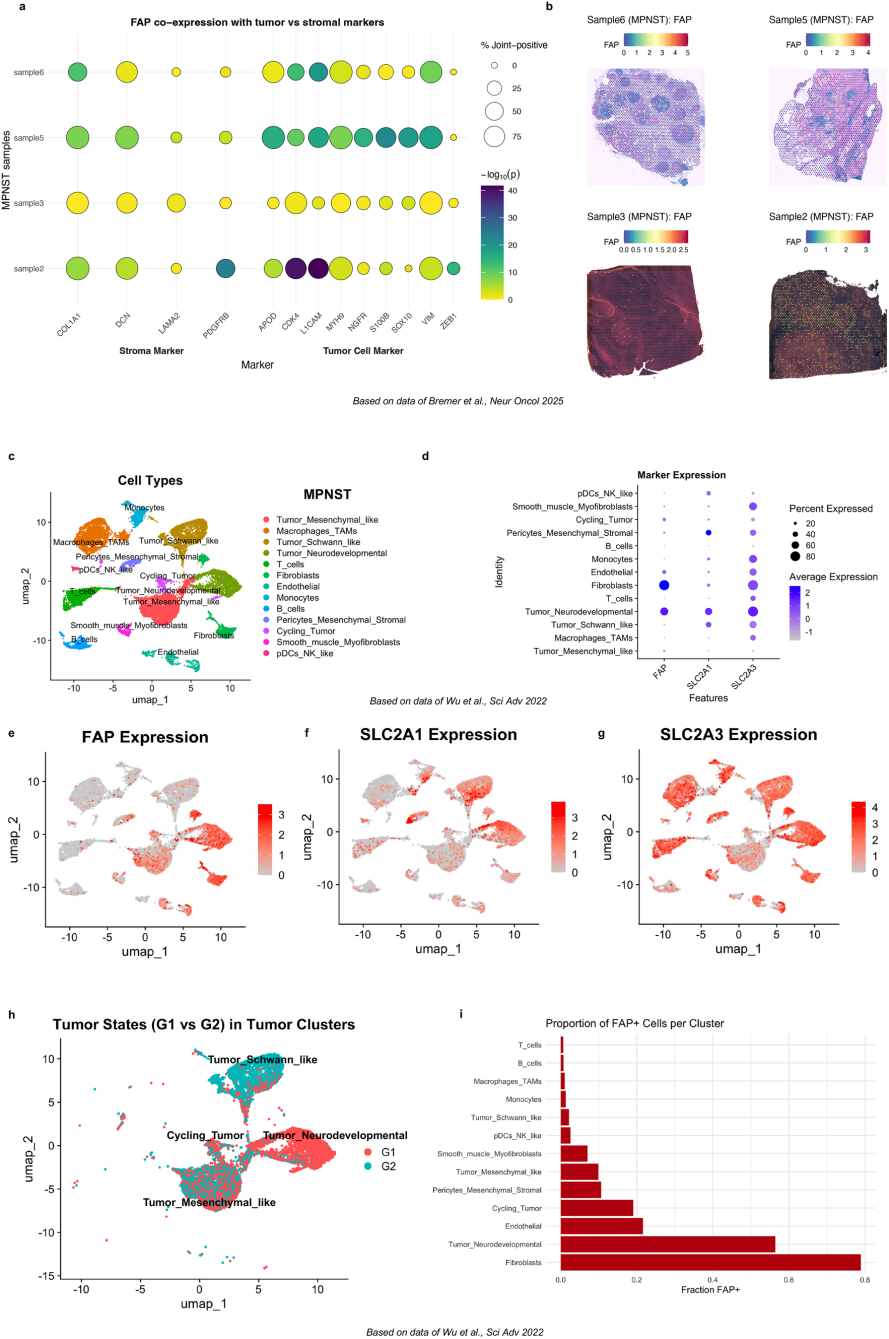
Spatial and single-cell resolution of *FAP* gene expression in MPNST. a FAP is co-expressed with several MPNST tumor cell markers in spatial transcriptomic data (dataset published by *Bremer *et al*.* in 2025) but also shows a relevant co-expression with canonical stroma markers. b Spatial feature plots of *FAP* gene expression across four MPNST samples, displaying relevant but heterogeneous *FAP* gene expression. c UMAP of 22 664 cells of four human MPNST samples, profiled by the 10× Genomics platform (dataset published by *Wu *et al. in 2022). d Dot plot showing *FAP*, *SLC2A1* and *SLC2A3* gene expression across the different cell types. *FAP* is predominantly expressed in tumor cells with a Schwann-like phenotype. e–g UMAPs displaying *FAP, SLC2A1* and *SLC2A3* expression across the different cell clusters. h UMAP of tumor cells assigned to MPNST-G1 (neural crest cell differentiation) and MPNST-G2 (Schwann cell-like precursor differentiation) based on the expression of different marker genes. For MPNST-G1: *TWIST1, SOX9, SNAI2, OTX2, PAX3, PAX6, SMO*. For MPNST-G2: *GAP43, PLP1, NGFR, PTCH1, WNT11*. i Barplot with the proportion of FAP-positive cells per cluster (threshold 0.5; for distribution of FAP expression, see Supplementary Fig. 6)

Spatial transcriptomics data [35] revealed an enrichment of spots with high *FAP, SLC2A1*, and *SLC2A3* expression in MPNST compared to non-MPNST lesions (Fig. 2g-i). *FAP*-high spots exceeded here the relative enrichment in MPNSTS of both *SLC2A1* and *SLC2A3*, supporting *FAP* as the most robustly differentially expressed marker and thereby suggesting potential diagnostic utility beyond metabolic readouts ([^18^F]FDG-PET/CT). *FAP* was consistently co-expressed with tumor cell markers across MPNST samples, while also present in stromal compartments as visualized by spatial co-expression mapping (Fig. 3a/b). *FAP* showed significant overlap with markers, such as *CDK4*, *SOX10*, and *L1CAM*, indicating distinct expression on tumor cells.

We could validate these findings in scRNA-seq data of four MPNSTs [38] proving that *FAP* is highly expressed in MPNST tumor cells but also in cancer-associated fibroblasts. *FAP* expression seems to be more specific to tumor cells compared to the surrounding microenvironment than *SLC2A3* expression (Fig. 3c-g and Supplementary Figure S3–6). Nevertheless, scRNA data reveal a differential *FAP* expression across different MPNST tumor cell states, indicating potential intra- and inter-tumoral heterogeneity (Fig. 3d) that we also observed in the spot-based spatial gene expression data (Fig. 3b). Higher *FAP* expression is here linked to a neural-crest lineage differentiation, previously described as the MPNST-G1 subgroup (Fig. 3h/i and Supplementary Figure S7). The MPNST-G1 subgroup, which is defined by sonic hedgehog (SHH) pathway activation and neural crest-like cell states, is associated with the poorest overall survival among MPNST patients [45]. Using Slingshot pseudotime [39] to model differentiation from neural crest-like (MPNST‑G1-like) to Schwann cell precursor-like (MPNST‑G2-like) states, we observed higher *FAP* expression in earlier neural crest-like tumor cells than in cells resembling the Schwann cell precursor-like state. This inverse relationship between pseudotime and *FAP* (Spearman’s ρ ≈-0.64, *p* < 0.001, Supplementary Figure S8) can be regarded as a potential hint supporting our hypothesis that *FAP* upregulation is a feature of cellular dedifferentiation, warranting further functional validation. Additionally, we observed that *FAP* expression in MPNST tumor cells correlates to some degree with an EMT-like (epithelial–mesenchymal-like) gene program *(even though EMT is primarily studied in the context of carcinomas, similar gene programs leading to increased invasiveness are described for sarcomas)*, especially within the aggressive neural crest-like subgroup, which is consistent with findings from other entities where *FAP* has been shown to promote EMT and tumor progression (Supplementary Figure S9/10) [46, 47].

Additionally, the scRNA data demonstrate that *FAP* expression is, as expected, inversely associated with 9p21 locus genes *(CDKN2A, CDKN2B, MTAP),* while some subsets of *FAP*-high tumor cells retain at least partial 9p21 expression, indicating that *FAP* upregulation may mark malignant tumor states beyond strict *CDKN2A/B* loss and could serve as a ‘broader’ malignancy marker; however, due to the limited sample size of the scRNA dataset, this should be further investigated in future approaches (Supplementary Figure S11).

### Immunohistochemistry confirms FAP upregulation in MPNSTs compared to neurofibromas

**Fig. 4:**
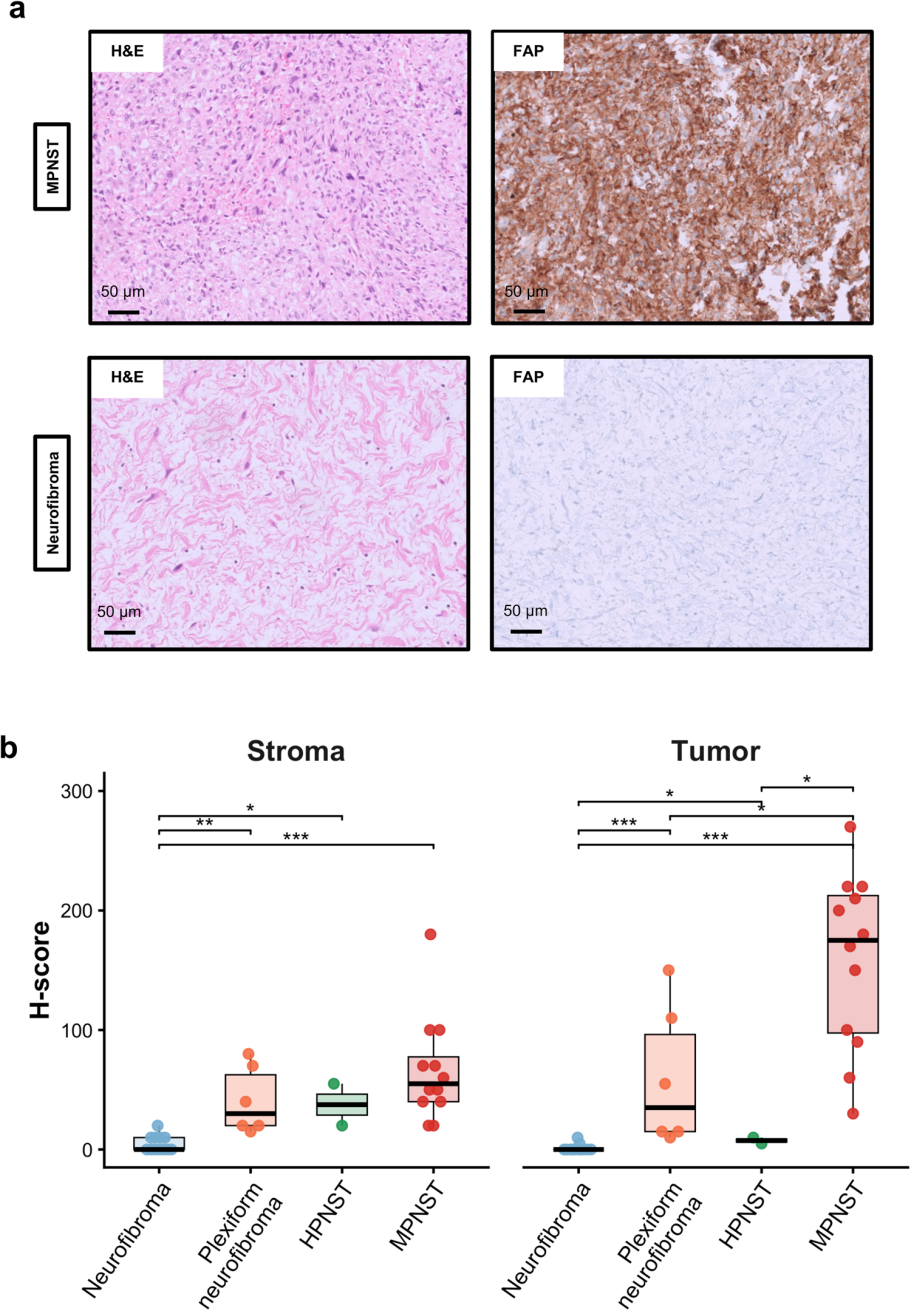
Immunohistochemical analysis confirms elevated FAP protein expression in MPNSTs relative to neurofibromas. a Representative H&E histology as well as FAP immuno-histochemical stains of MPNST (top row) and neurofibroma cases (bottom row). MPNSTs are characterized by pleomorphic, hyperchromatic spindle cells with brisk mitotic activity, and strong membranous and cytoplasmic FAP expression. On the contrary, neoplastic Schwann cells in neurofibromas are characterized by bland spindle cell morphology with thin, wavy nuclei and myxoid to collagenous stroma with absent to low FAP expression. b H scores for immuno-histochemical FAP expression in MPNSTs (*n* = 12), conventional neurofibromas (*n* = 12), plexiform neurofibromas (*n* = 6) and hybrid peripheral nerve sheath tumors (HPNSTs, *n* = 2) shown for tumor and stromal compartments. MPNSTs exhibited significantly higher FAP expression compared to neurofibromas in stromal as well as tumor cells (both *p* < 0.001, Wilcoxon rank-sum test). p value significance is indicated as follows: *p* < 0.05 *, *p* < 0.01 **, *p* < 0.001 ***. Only statistically significant pairwise comparisons are indicated in the plot. Exact p values and corresponding multiple-testing adjusted q values for all comparisons are provided in Table S2

Even though there is some evidence that MPNSTs show relevant FAP expression upon immunohistochemistry [48], no prior IHC study has systematically assessed FAP as a discriminatory marker between malignant and benign peripheral nerve sheath tumors, which comprise rather rare tumor entities. Therefore, we performed FAP-IHC on archival human tissue samples of MPNSTs (*n* = 12) as well as conventional neurofibromas (*n* = 12; median age of MPNST patients [including range]: 31 (16–86); median age of neurofibroma patients [including range]: 48 (10–69); male/female ratio MPNST patients 7/5; male/female ratio neurofibroma patients 8/4). MPNSTs showed moderate to strong FAP expression on most tumor cells and partly also in surrounding stromal cells (Fig. 4a, and Supplementary Figure S12a/b). Hence, MPNSTs displayed significantly higher FAP expression in the tumor as well as stroma compartment than neurofibromas (Fig. 4b, both *p* < 0.001). In some neurofibromas, a faint, partly perilesional FAP expression was seen (Supplementary Figure S12c/d). Interestingly, despite a generally strong FAP expression in MPNSTs, IHC confirmed the inter- and intra-tumoral heterogeneity of FAP expression in MPNST tumor cells, which we also observed in spatial and scRNA data (Fig. 3b/d, and Supplementary 12b). To obtain a more comprehensive view of FAP expression in peripheral nerve sheath tumors, we also investigated FAP expression in six plexiform neurofibromas, which have a higher potential of malignant transformation than conventional neurofibromas, and in two hybrid peripheral nerve sheath tumors (Supplementary Figure S13). Interestingly, plexiform neurofibromas exhibited higher FAP expression than conventional neurofibromas both in the perilesional stroma (*p* = 0.001) and tumor cells (*p* < 0.001). Nevertheless, MPNSTs displayed higher FAP expression in tumor cells than plexiform neurofibromas (*p* = 0.015).

Results of immunohistochemical studies are displayed in Fig. 3b and in Table S2 (with unadjusted *p * and adjusted *q* values).

Notably, a careful histopathologic review of all H&E and corresponding FAP-stained sections revealed instructive intra-tumoral patterns supporting a stepwise association of FAP expression with malignant transformation in two cases: In one NF-1-associated case, a benign neurofibroma directly adjacent to an MPNST showed absent FAP expression, while the MPNST displayed strong membranous and cytoplasmic FAP expression (Supplementary Figure S14). In a second NF-1-related MPNST, areas with high cellularity, nuclear atypia, and increased mitotic activity exhibited markedly strong FAP expression compared with adjacent regions of lower cellularity and minimal atypia, which likely represent residual or transitioning neurofibroma-like components (Supplementary Figure S15).

We observed almost no relevant immunoreactivity in neurofibromas and uninvolved healthy tissue (including peripheral nerves) except of eccrine sweat glands, which are known to express dipeptidyl peptidase IV (DPP4), a serine protease that is closely related to FAP with a high structural similarity (BLAST alignment revealed 51.9% sequence identify over 98% query coverage between human FAP and DPP4, see Supplementary Figure S16) [49]. Hence, low-level cross-reactivity in high DPP4 expressing tissues may explain the isolated signal observed in eccrine glands and should be considered when interpreting FAP immunohistochemistry.

To further validate staining specificity, we consistently observed strong FAP expression in cancer-associated fibroblasts in multiple solid carcinomas, serving as an internal positive control. Representative examples, including metastatic colorectal and pancreatic ductal adenocarcinoma, are provided in Supplementary Figure S17 and demonstrate robust stromal but absent or minimal tumor cell-intrinsic FAP expression, in line with prior reports [50] and consistent with our previous work [13]. In addition, perivascular stromal FAP expression adjacent to MPNSTs was frequently observed (Supplementary Figure S18).

### Clinical validation of FAP as a malignancy biomarker using PET/CT in an NF-1 patient

**Fig. 5:**
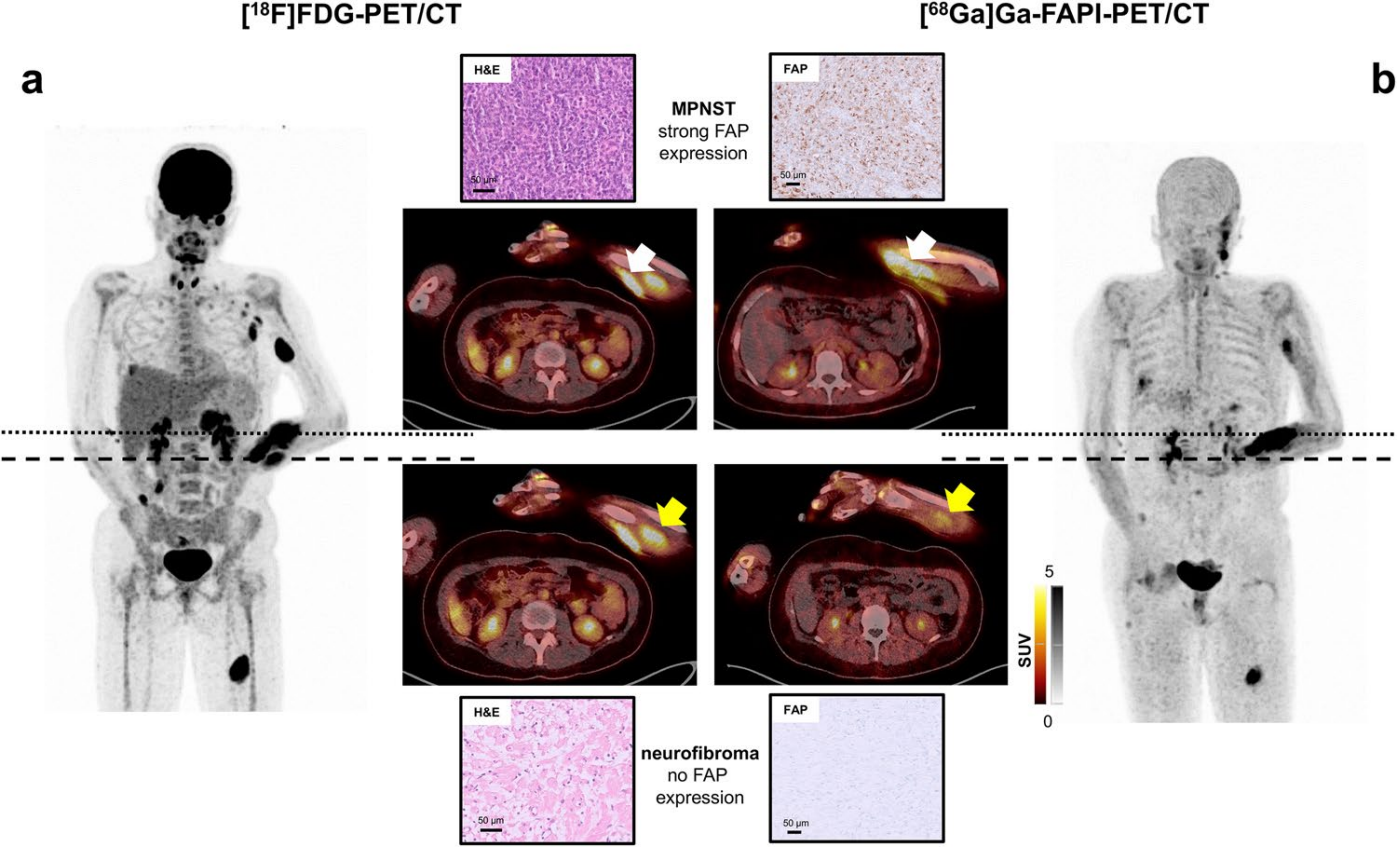
Molecular imaging of FAP as a malignancy biomarker using [⁶⁸Ga]Ga-FAPI PET/CT in a NF-1 patient. Maximum intensity projections and transaxial slices of [^18^F]FDG (**a**) and [^68^ Ga]Ga- LNTH-1363S (**b**) PET/CT. A neurofibroma in the left forearm (dashed line; yellow arrow) shows high [^18^F]FDG uptake (**a**) and FAP expression (**b**) uptake (SUV_max_ 6.8 vs. 3.5). In contrast, the MPNST on the left forearm (dotted line; white arrow) shows high [^18^F]FDG uptake (**a**) and high FAP expression (**b**) (SUV_max_ 10.7 vs. 14.7). Histological analysis revealed the typical morphology of a bland looking neurofibroma with no FAP protein expression upon immunohistochemistry in the lesion adjacent to the median nerve, whereas the MPNST lesion on the left forearm consisted of a hyper-cellular tumor with hyperchromatic, mitotically active spindle cells with strong cytoplasmic and membranous FAP expression. Noteworthy, hypermetabolic, FAP-negative axillary lymph nodes could be confirmed as reactive secondary to superimposed infection of the ulcerating sarcoma

Building on our transcriptomic and immunohistochemical findings, we next asked whether FAP expression could be leveraged for non-invasive detection of malignancy in NF-1 patients. A 31-year-old NF-1 patient with suspected malignant transformation in a rapidly enlarging mass in the left forearm was transferred to our Department of Nuclear Medicine for further imaging. The patient received molecular imaging with FAP-directed and [^18^F]FDG-PET/CT, demonstrating various tracer-avid lesions in both examinations. The rapidly enlarging mass on the left forearm presented with intense FAP expression and [^18^F]FDG-avidity and was histopathologically confirmed as malignant sarcoma. A FAP- and [^18^F]FDG-positive lesion in the right pleura was also identified as sarcomatous transformation (Fig. 5). In contrast, another lesion on the left forearm adjacent to the left median nerve showed suspiciously high glucose metabolism but no relevant FAP expression. It was histopathologically confirmed as a benign neurofibroma (Fig. 5). In addition, multiple hypermetabolic, FAP-negative axillary lymph nodes could be proven as reactive secondary to a superimposed infection of the ulcerating sarcoma in the forearm. Noteworthy, a lesion in the left thigh which showed patchy tracer accumulation (SUV_max_ (FDG): 14.3, SUV_max_ (FAP): 9.5) was identified as a neurofibroma with no signs for malignant transformation.

## Discussion

Identifying reliable molecular markers for malignant transformation in NF-1 is critically important, as early and accurate distinction between benign neurofibromas and MPNSTs remains a major clinical challenge with direct implications for prognosis, surveillance, and therapeutic decision-making.

By investigating multiple publicly available bulk, spatial, and single-cell transcriptomic datasets, we provide strong evidence for an upregulation of *FAP* gene *e*xpression in MPNSTs compared to neurofibromas. We could show that there is a low *FAP* gene expression in neurofibromas on RNA level, which is consistent with the findings of *Brosseau *et al*.* showing that the expression of classic fibrogenic markers, such as *FAP* or *COL11A1* is low in neurofibromas based on single-cell RNA data [51]. On the contrary, our analyses demonstrate strong FAP expression on gene as well as protein level in MPNSTs. *Beer *et al*.* also could show in their immunohistochemical study that almost all MPNSTs show strong FAP protein expression [48]. Previously, it has been demonstrated that uptake of FAP-directed PET vectors and the histopathologic FAP expression correlate substantially [22].

The limited specificity of [^18^F]FDG-PET in NF-1 likely reflects, in part, the elevated glucose metabolism observed in certain benign lesions such as hybrid neurofibroma/perineurioma tumors, which occur almost exclusively in NF-1 and show strong GLUT1 expression on immunohistochemistry, which serves here even diagnostic purposes for the perineurioma component [52]. We propose that this GLUT1-driven metabolic activity in perineurioma components may underlie the FDG avidity of some histologically benign lesions in NF-1, thereby confounding non-invasive diagnosis of tumor transformation. In contrast, our data support FAP as a more malignancy-specific biomarker.

Our spatial and single-cell transcriptomic analyses consistently localize *FAP* expression to both malignant tumor cells and CAFs within MPNSTs, which was validated by immunohistochemistry and is line with prior reports on FAP protein expression in other sarcoma subtypes [11]; notably, single-cell resolution reveals that *FAP* is particularly enriched in the MPNST-G1 subtype, characterized by a neural crest-like, more primitive phenotype, SHH pathway activation and poor clinical outcomes, highlighting a novel and potentially actionable marker in this aggressive subgroup [45]. This finding not only expands the therapeutic relevance of FAP beyond stromal targeting but also underscores the cellular and molecular heterogeneity that defines MPNST biology and should inform future stratified treatment approaches.

While our data support FAP as a marker of malignant transformation, isolated case reports have described benign lesions, such as fibromas or schwannomas, with moderate FAPi-uptake likely due to stromal fibrosis [53, 54]. Nevertheless, in a neurofibromatosis patient with pleomorphic rhabdomyosarcoma, malignant lesions demonstrated markedly higher FAPi-uptake than benign cutaneous fibromas, consistent with our findings [53].

While FAP-directed PET/CT demonstrated excellent concordance with histologically confirmed malignancy in our index case, one neurofibroma located at the sciatic nerve, that exhibited moderate, patchy tracer uptake, lacked any detectable FAP protein expression in immunohistochemistry. This discrepancy and the patchy tracer accumulation in PET imaging may reflect localized stromal remodeling (we observed FAP expression in perilesional fibroblasts in neurofibromas, see Supplementary Fig. 12d), or areas of dedifferentiation not sampled by the incisional biopsy - phenomena previously described in a previous case report by *Wu *et al*.* without showing histological or immuno-histochemical staining [53]. Such findings underscore the need for integrative imaging-pathology correlation and highlight the importance of validating FAP-directed PET/CT in larger NF-1 cohorts to refine its specificity in clinical practice.

Given the role of atypical neurofibromatous neoplasms of uncertain biological potential (ANNUBP) as an intermediate stage in the progression from plexiform neurofibromas to MPNST [8], future studies should investigate FAP expression in these lesions and assess its correlation with malignant transformation risk and clinical progression, as our cohort currently did not comprise any ANNUBPs.

While FAP expression is not specific to MPNST and has been reported in other sarcoma entities, such as undifferentiated pleomorphic sarcoma (UPS) or myxofibrosarcoma [11], its upregulation may be a general feature of malignant transformation. Our data demonstrate a clear increase of FAP expression in malignant peripheral nerve sheath tumors compared with benign neurofibromas, which represents the most clinically relevant distinction in the context of NF-1-associated tumor surveillance. In the diagnostic context of peripheral nerve sheath tumors, established biomarkers, such as H3K27me3 loss and p16 inactivation, provide important but imperfect information, as H3K27me3 loss, while frequent in sporadic and radiation-associated MPNSTs, is less consistent in NF-1-related tumors [55] and not entirely specific [56]. Loss of p16 may occur early even in benign neurofibromas [56]. In contrast, although FAP upregulation is not specific to MPNST and is observed across high-grade sarcomas [11], its major added value lies in its theranostic potential, offering a biologically meaningful, non-invasive imaging read-out (e.g., FAP-targeted PET/CT) that may enable early detection and monitoring of malignant transformation in NF-1 patients beyond what conventional tissue-based markers can provide.

Thus, irrespective of histogenetic specificity, FAP upregulation serves as a biologically meaningful marker of malignancy in this context.

Importantly, although comprehensive DNA methylation profiling could only be successfully performed in a subset of cases, all tumors underwent an extensive diagnostic work-up including detailed histomorphology, broad immunohistochemical panels, clinical correlation, and RNA-based fusion profiling to exclude relevant mimics. Taken together, this multimodal diagnostic framework provides a robust basis for our conclusions. Future prospective studies nevertheless should systematically incorporate methylation profiling [28], especially given the recent methylation classifier expansions (v12.8) specifically including plexiform neurofibromas [57], which will further refine molecular classification but is unlikely to alter the central observation of FAP upregulation as a biologically meaningful marker of malignant transformation in peripheral nerve sheath tumors.

The general feasibility of the clinical translation of our tissue-based results could be demonstrated in a single patient with NF-1 in whom FAP-directed imaging provided a more accurate non-invasive in vivo characterization of multiple tumor lesions as compared to FDG-PET/CT. Future studies to further investigate the values of FAP-directed PET/CT as a reliable read-out of tumor malignancy are highly warranted.

Hence, our findings highlight FAP as not only a diagnostic marker distinguishing MPNST from benign NF-1-associated lesions, but also a rational therapeutic target, which is clinically important considering the limited therapeutic options and the dismal prognosis of MPNST patients [3].

Radiopharmaceutical therapies using FAP ligands have shown early clinical and pre-clinical promise in sarcomas [18, 20, 50, 58]. Notably, several MPNSTs in our cohort exhibited FAP protein expression levels comparable to those recently reported in solitary fibrous tumors treated successfully with FAPi-targeted radiopharmaceutical therapy, underscoring the biological plausibility and translational feasibility of FAP profiling and FAP-targeted theranostic approaches in MPNST [58].

On top of that, pre-clinical studies have demonstrated that FAP-targeted radiopharmaceutical therapy can synergize with immune checkpoint inhibitors, enhancing antitumor efficacy [25, 50, 59], which could pave the way to new combinatory treatment strategies in MPNST [60].

By systematically integrating high-throughput transcriptomic analyses, protein-level validation, and clinical molecular imaging, we demonstrate that FAP is consistently upregulated in malignant peripheral nerve sheath tumors (MPNSTs) compared to neurofibromas. This is the first study to comprehensively characterize FAP expression across bulk, spatial, and single-cell resolution on transcriptome and protein level in NF-1-associated tumors. The successful translation of these molecular insights into clinical whole-body imaging in an NF-1-patient index case underscores FAP’s potential as a non-invasive biomarker of malignancy. These findings support the further development of FAP-based imaging and therapies in sarcoma care, particularly for NF-1 patients.

## Supplementary Information

Below is the link to the electronic supplementary material.

Supplementary file1 (PDF 8362 KB)

## Data Availability

All analysis scripts used in this study are available on GitHub (https://github.com/ngr-path/Analysis_R_FAP_NF1_MPNST). Publicly available datasets referenced in the manuscript can be accessed via their respective repositories, as cited. Immunohistochemistry and clinical imaging data are available from the corresponding authors (NGR, CL) upon reasonable request.
