# Supplementary material for: FAP expression as a marker of malignant transformation enabling in vivo characterization in peripheral nerve sheath tumors: a multimodal and translational study
Source: Acta Neuropathol. 2026 Jan 27;151(1):11. doi: 10.1007/s00401-026-02979-7 (PMC12847211; doi:10.1007/s00401-026-02979-7)
Supplement: Supplementary file 1 — Supplementary file1 (PDF 8362 KB) [file 401_2026_2979_MOESM1_ESM.pdf]

Supplementary Material (Figures & Tables) for

FAP Expression as a Marker of Malignant Transformation Enabling In-Vivo Characterization in Peripheral Nerve Sheath Tumors: A Multimodal and Translational Study

Short Title: FAP in peripheral nerve sheath tumors

Nic G. Reitsam[1,2,3*], Alexander Gäble[4], Lisa Siebenhüter[1], Tina Schaller[1,3,5], Friederike Liesche-Starnecker[1,3,5,6,7], Eva Sipos[1], Sebastian Dintner[1,3], Christoph Walz[8], John Babic[9], Martin Trepel[3,5,10], Malte Kircher[2,3], Victoria E. Fincke[3,11], Pascal D. Johann[3,11], Bruno Märkl[1,3,5], Constantin Lapa[3,4,5,*], Johanna S. Enke[3,4]

[1] Pathology, Faculty of Medicine, University of Augsburg, Augsburg, Germany.
[2] Else Kroener Fresenius Center for Digital Health, Technical University Dresden, Dresden, Germany.
[3] Bavarian Cancer Research Center (BZKF), Augsburg, Germany.
[4] Nuclear Medicine, Faculty of Medicine, University of Augsburg, Augsburg, Germany.
[5] Comprehensive Cancer Center, Faculty of Medicine, University of Augsburg, Augsburg, Germany.
[6] Department of Neuropathology, Pathology, University of Augsburg, Augsburg, Germany.
[7] Institute of Neuropathology, University Medical Center Ulm, Faculty of Medicine, Ulm University, Ulm, Germany
[8] Institute of Pathology, Faculty of Medicine, LMU Munich, Munich, Germany
[9] Ratio Therapeutics, Boston, Massachusetts.
[10] Hematology and Oncology, Faculty of Medicine, University of Augsburg, Augsburg, Germany.
[11] Pediatrics and Adolescent Medicine, Swabian Children's Cancer Center, University Hospital Augsburg, Germany.

Key Words: Peripheral Nerve Sheath Tumors; Neurofibromatosis Type 1; FAP; Fibroblast Activation Protein; Molecular Imaging; PET; MPNST

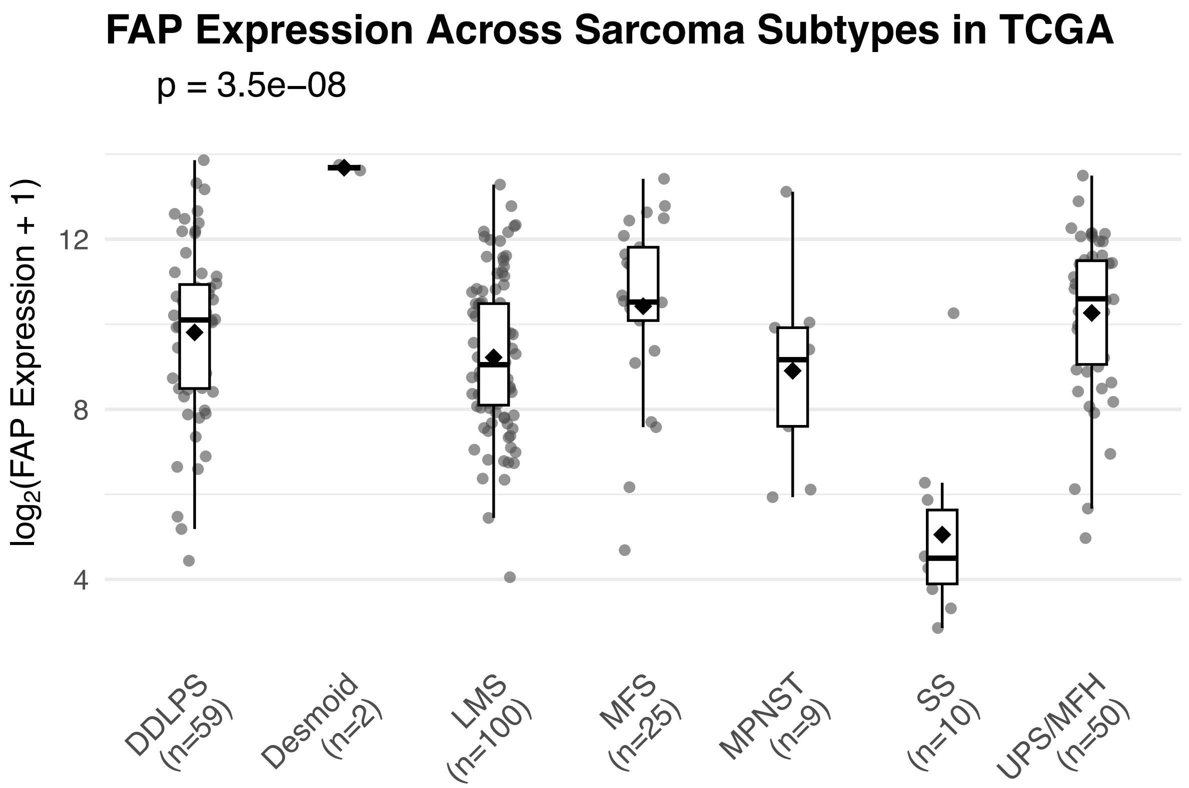

Supplementary Figure S1. ***FAP*** **mRNA expression across soft tissue tumor subtypes in TCGA.** Log_2-transformed FAP expression levels (RSEM + 1) are shown for seven sarcoma subtypes from the TCGA SARC cohort (n = total 255, TCGA PanCancer Atlas). There was a differential *FAP* gene expression across subtypes ($p < 0.001$). Notably, Synovial Sarcoma exhibited the lowest FAP expression, while MPNST, LMS, MFS, DDLPS and UPS/MFH showed comparably higher levels. Desmoid tumors showed very high *FAP* expression, though only two samples were available for this subtype.

Data are presented as individual samples (jittered points), with boxplots representing median and interquartile range, and black diamonds indicating group means

Tumor subtypes: DDLPS = Dedifferentiated Liposarcoma, LMS = Leiomyosarcoma, MFS = Myxofibrosarcoma, UPS/MFH = Undifferentiated Pleomorphic Sarcoma/Malignant Fibrous Histiocytoma, SS = Synovial Sarcoma, MPNST = Malignant Peripheral Nerve Sheath Tumor.

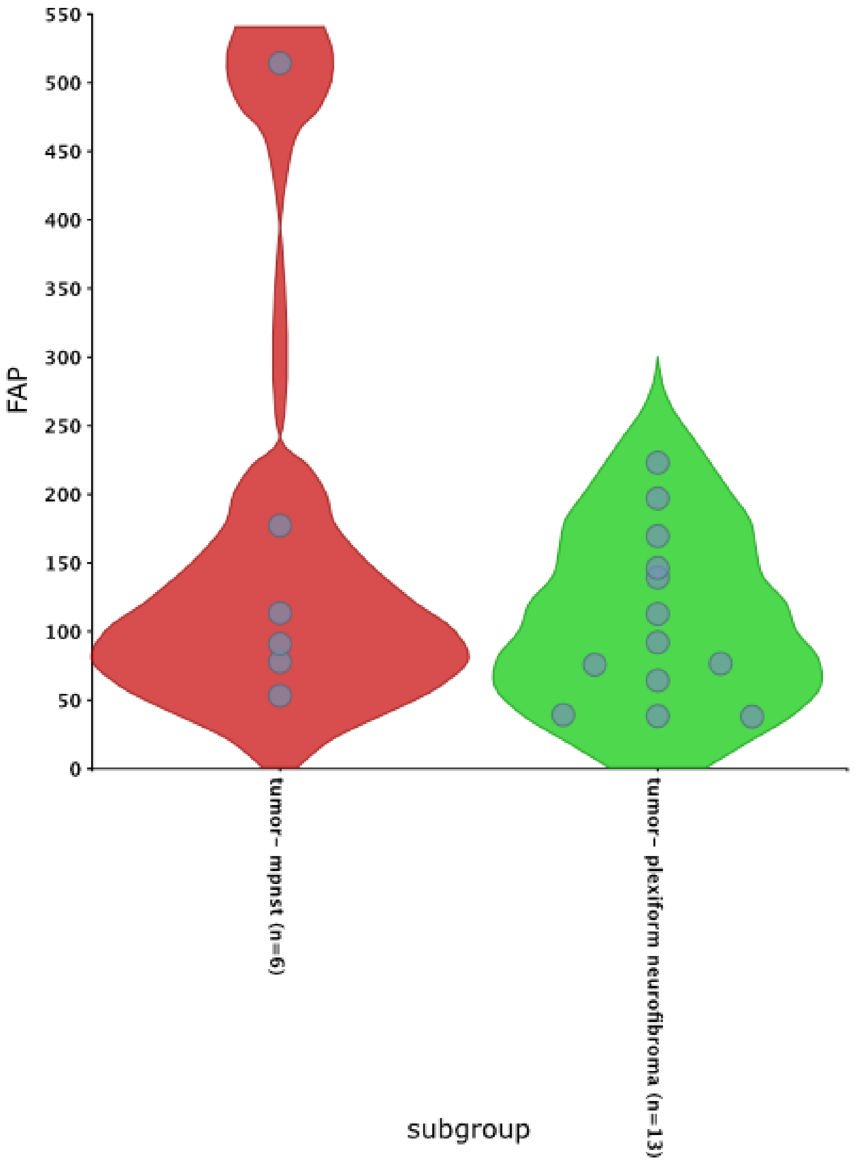

Supplementary Figure S2. FAP gene expression in plexiform neurofibromas and MPNSTs in *Miller et al., EMBO Molecular Medicine 2009* dataset. We note that in a limited dataset reported by *Miller et al.* (MPNST n = 6; plexiform neurofibroma n = 13), *FAP* expression appeared heterogeneous and in some cases at similar levels in plexiform neurofibromas and MPNSTs. Given the small sample size and variability, these data should be interpreted with caution but may suggest that *FAP* expression is not entirely specific to malignant transformation. Nevertheless, plexiform neurofibromas show the highest progression rate to MPNSTs and therefore higher *FAP* gene expression in plexiform than conventional neurofibromas does also support our hypothesis of *FAP's* association to increased malignant potential.

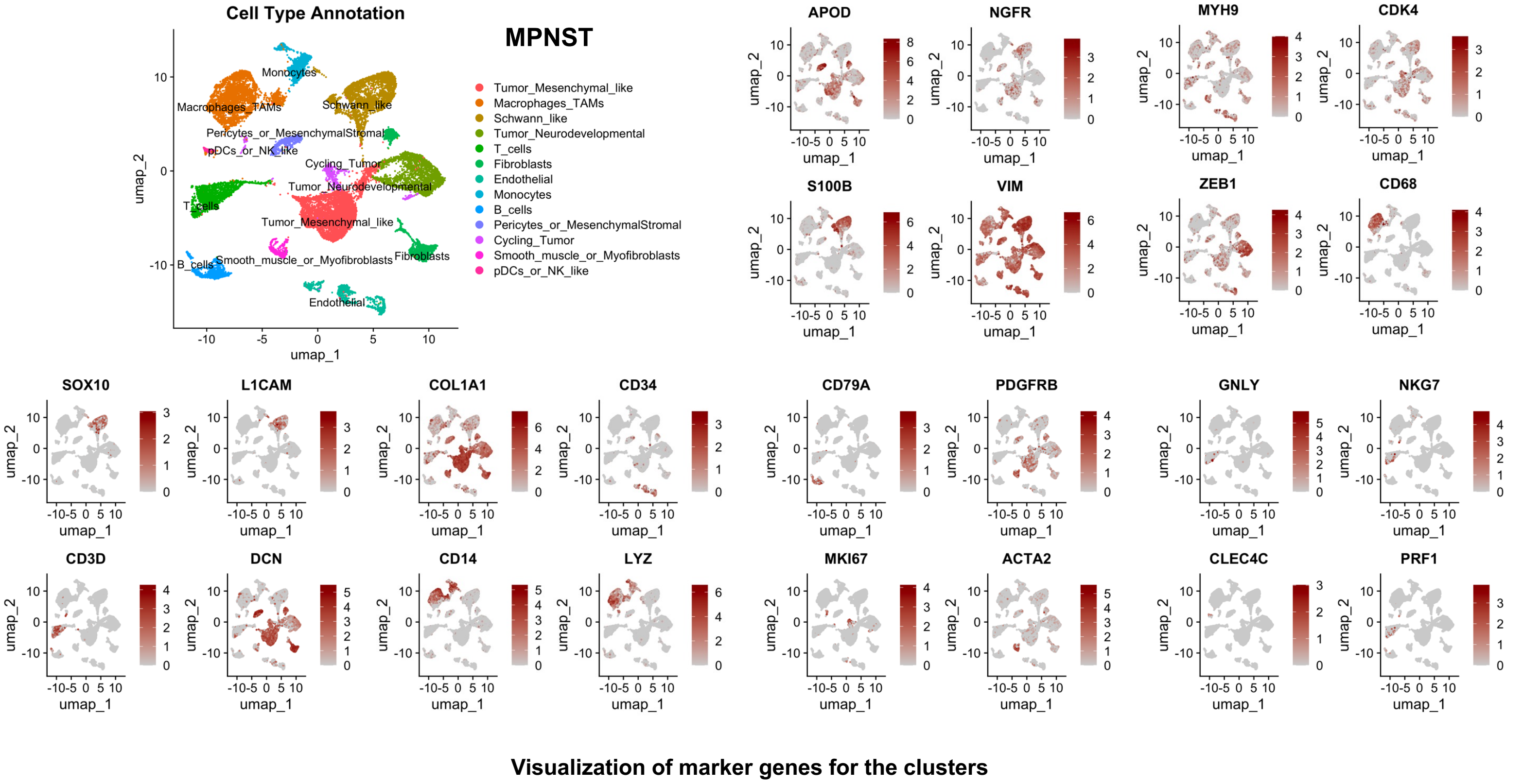

Supplementary Figure S3. Cell type annotation in the MPNST single cell RNA data (*n*=4) and visualization of marker genes for each cluster. Based on marker genes, we manually annotated cell types in the MPNST scRNA data. Different tumor cell clusters could be identified (Schwann-like tumor, mesenchymal-like tumor, neural-crest-like tumor [neurodevelopmental] and proliferative, cycling tumor). Several typical marker genes are here visualized for transparency of cluster labeling.

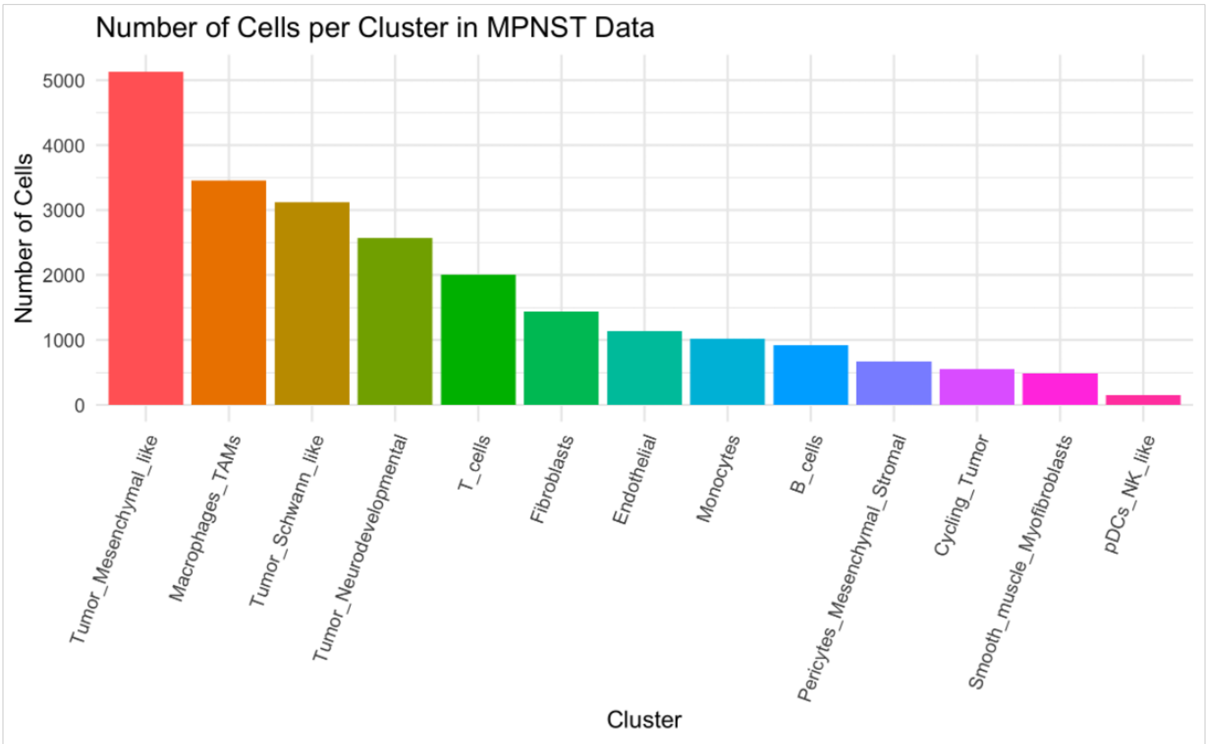

Supplementary Figure S4. Number of cells per cluster in single cell RNA MPNST data. As expected, tumor cells mark the predominant cell population in the MPNST single cell RNA data.

Cluster and number of cells: Tumor_Mesenchymal_like: 5134, Macrophages_TAMs: 3451, Tumor _Schwann_like 3123, Tumor_Neurodevelopmental 2565, T_cells 2006, Fibroblasts 1444, Endothelial 1141, Monocytes 1017, B_cells 923, Pericytes_or_MesenchymalStromal 670, Cycling_Tumor 552, Smooth_muscle_or_Myofibroblasts 482, pDCs_or_NK_like 156.

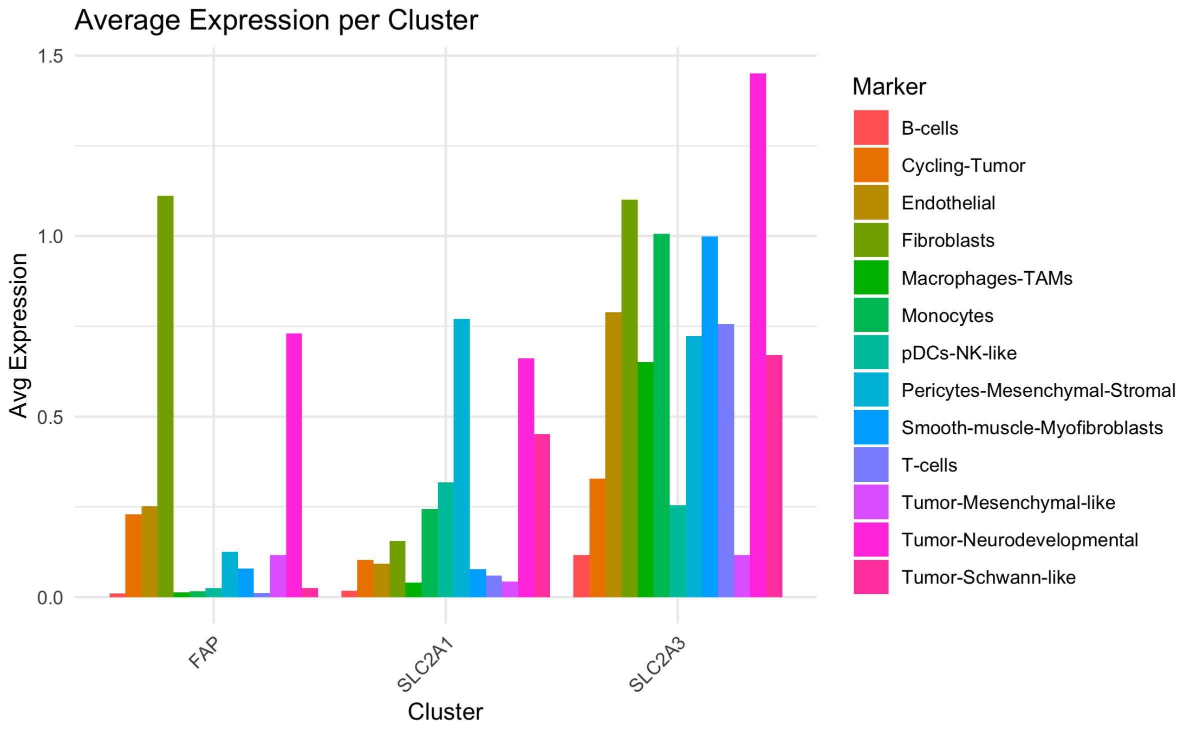

Supplementary Figure S5. Average Expression of *FAP*, *SLC2A1* and *SLC2A3* in single cell RNA data across different cell types/clusters. *FAP*, *SLC2A1* and *SLC2A3* gene expression vary between the different cell clusters. *FAP* is mainly expressed in fibroblasts and and so-called neurodevelopmental tumor cell subpopulations (with neural crest-like properties). *SLC2A3*, encoding for GLUT3, is expressed across several cell types. *SLC2A1*, encoding for GLUT1, is mainly expressed in the neural crest-like and Schwann-like tumor cells but also in stromal cells (pericytes).

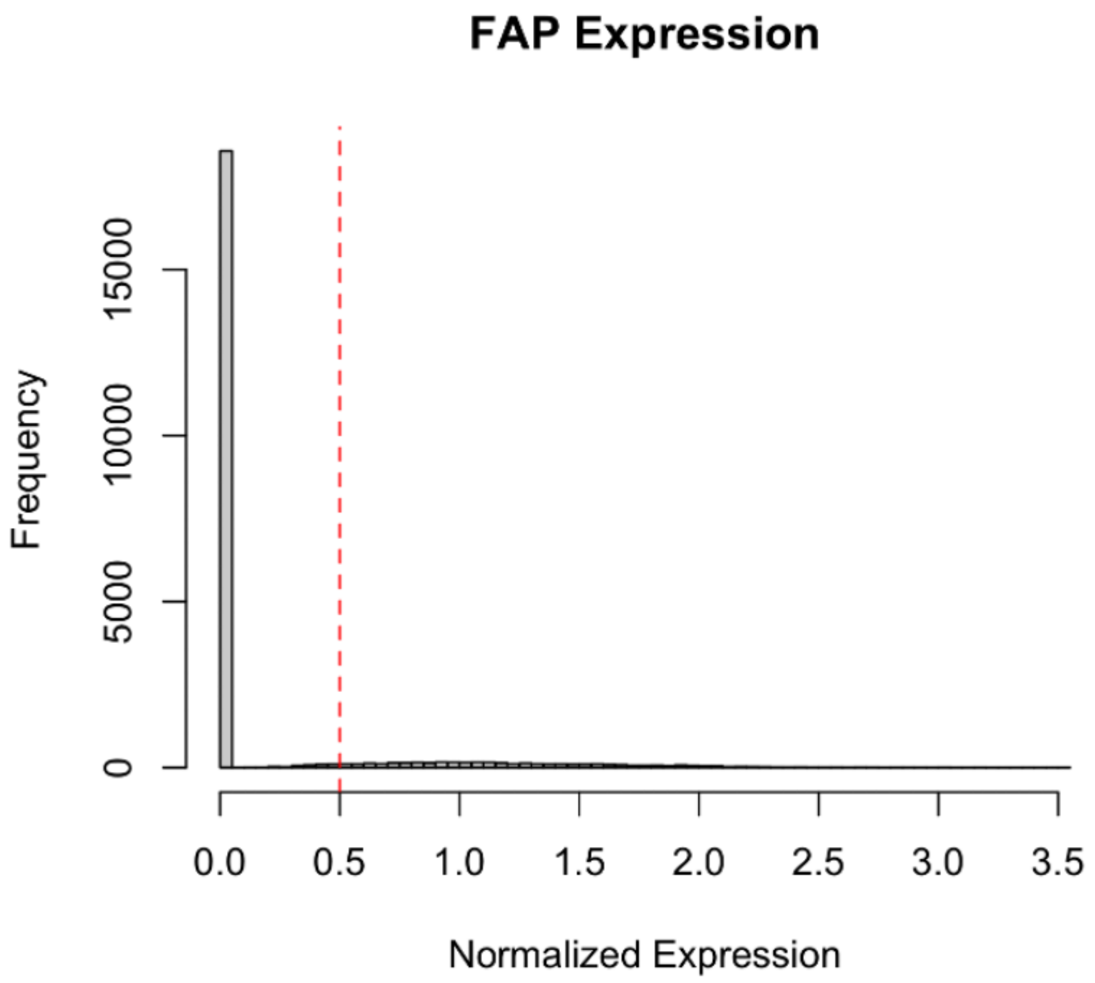

Supplementary Figure S6. Distribution of *FAP* expression values in single cell RNA MPNST data. Cut-off for FAP+ cells in Figure 3I was set at 0.5.

scRNA data represents zero-inflated sparse data.

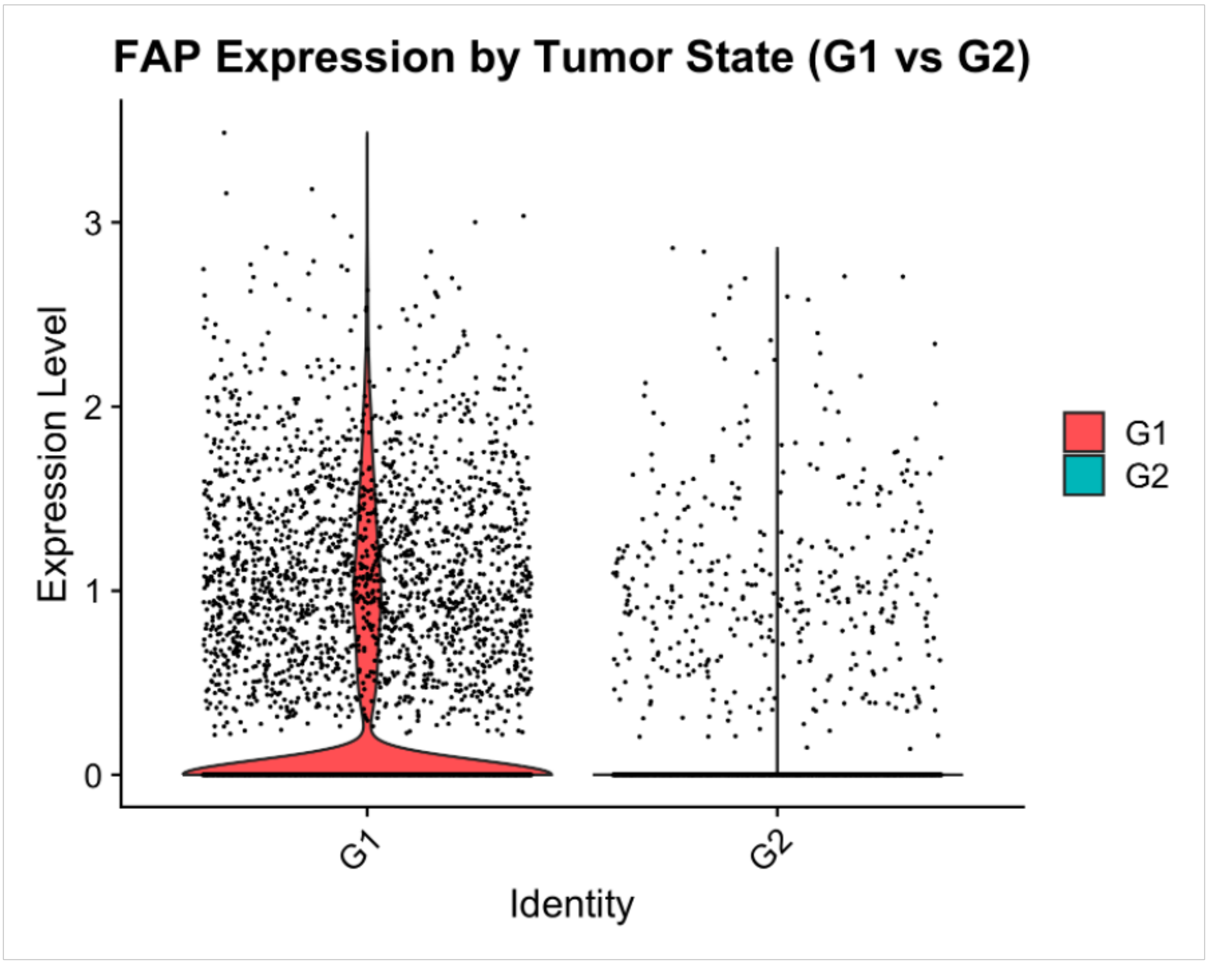

Supplementary Figure S7. *FAP* expression in single cell RNA MPNST data stratified by MPNST subgroup (G1 vs G2). MPNST-G1-like subgroup tumor cells show higher *FAP* expression than MPNST-G2-like subgroup tumor cells. MPNST-G1-like tumor cells have been shown to be associated with a more neural crest-like phenotype and a more aggressive behavior.

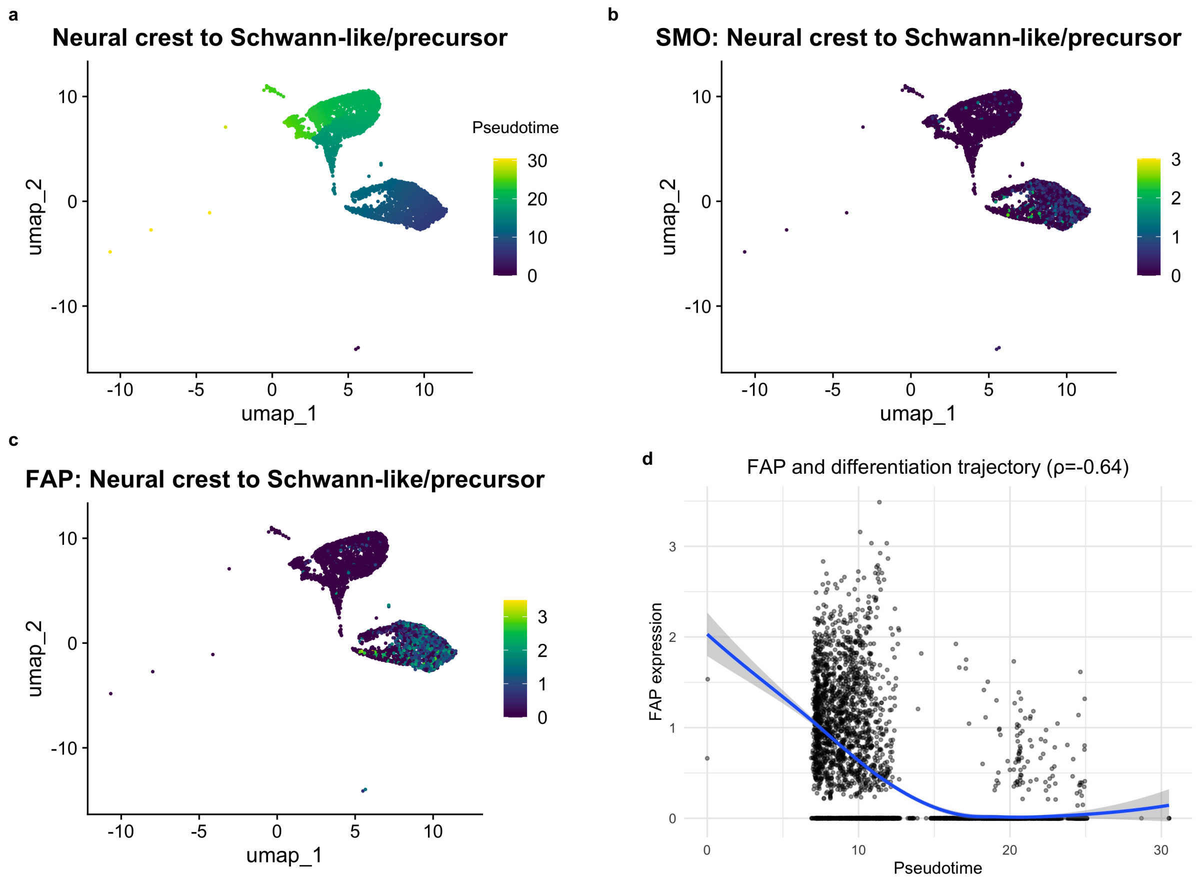

Supplementary Figure S8. *FAP* expression and pseudotime analysis. FAP expression decreases along the pseudotime trajectory from neural crest-like to Schwann-precursor-like tumor cells. **a** UMAP visualization of tumor cells colored by Slingshot-derived pseudotime, rooted in neural crests-like cells and progressing toward Schwann-precursor-like cells. The gradient illustrates a developmental continuum aligned with Schwann cell lineage progression. **b** UMAP visualization for *SMO* expression; *SMO* is involved in sonic hedgehog signaling and overexpressed in MPNST-G1 tumors. **c** UMAP visualization for *FAP* expression (higher in more primitive, neural crest-like tumor cells than in Schwann-like-precursor tumor cells). **d** Scatterplot showing FAP expression as a function of pseudotime. A strong negative correlation (Spearman's ρ = –0.64) indicates that *FAP* expression is highest in neural crest-like tumor cells and declines along the trajectory, supporting its association with a more primitive, dedifferentiated aggressive tumor state.

The mesenchymal-like tumor cells do not lie on the inferred trajectory; therefore, we did not include them here.

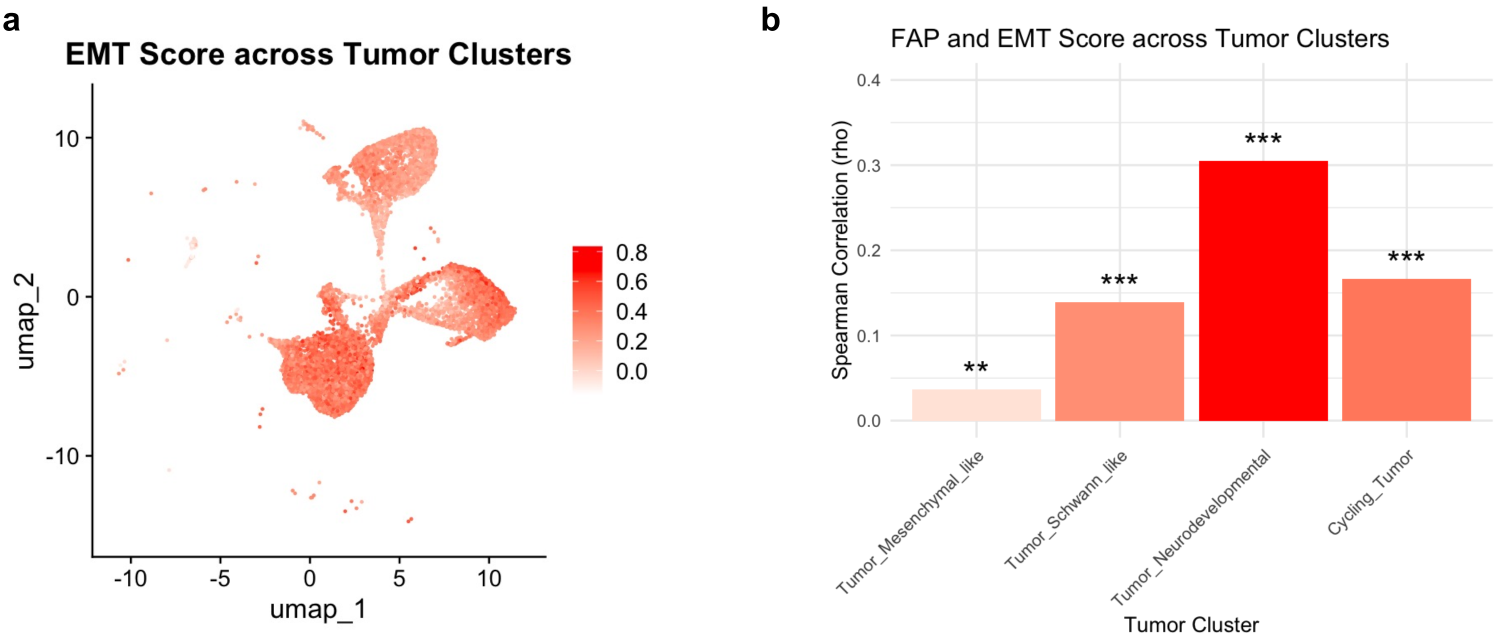

Supplementary Figure S9. FAP gene expression and EMT-like gene programs. a UMAP visualization of module score (*AddModuleScore* function in Seurat) of EMT gene set (https://www.gsea-msigdb.org/gsea/msigdb/cards/HALLMARK_EPITHELIAL_MESENCHYMAL_TRANSITION.html). **b** Spearman correlation between *FAP* and EMT-like score across tumor clusters (*** < 0.001, ** < 0.01). Strongest correlation between *FAP* expression and EMT-like gene score was observed in the neural crest-like (Tumor_Neurodevelopmental) cluster with a modest rho value of around 0.3, suggesting a potential relationship between *FAP* expression and EMT activity.

Although classical EMT is primarily described in epithelial cancers, sarcomas demonstrate similar cellular plasticity linked to invasiveness and dedifferentiation, consistent with emerging evidence in other tumor types.

Genes *GLT25D1, PRSS2* were not available in the processed dataset and hence were neglected for calculation of EMT-like module score.

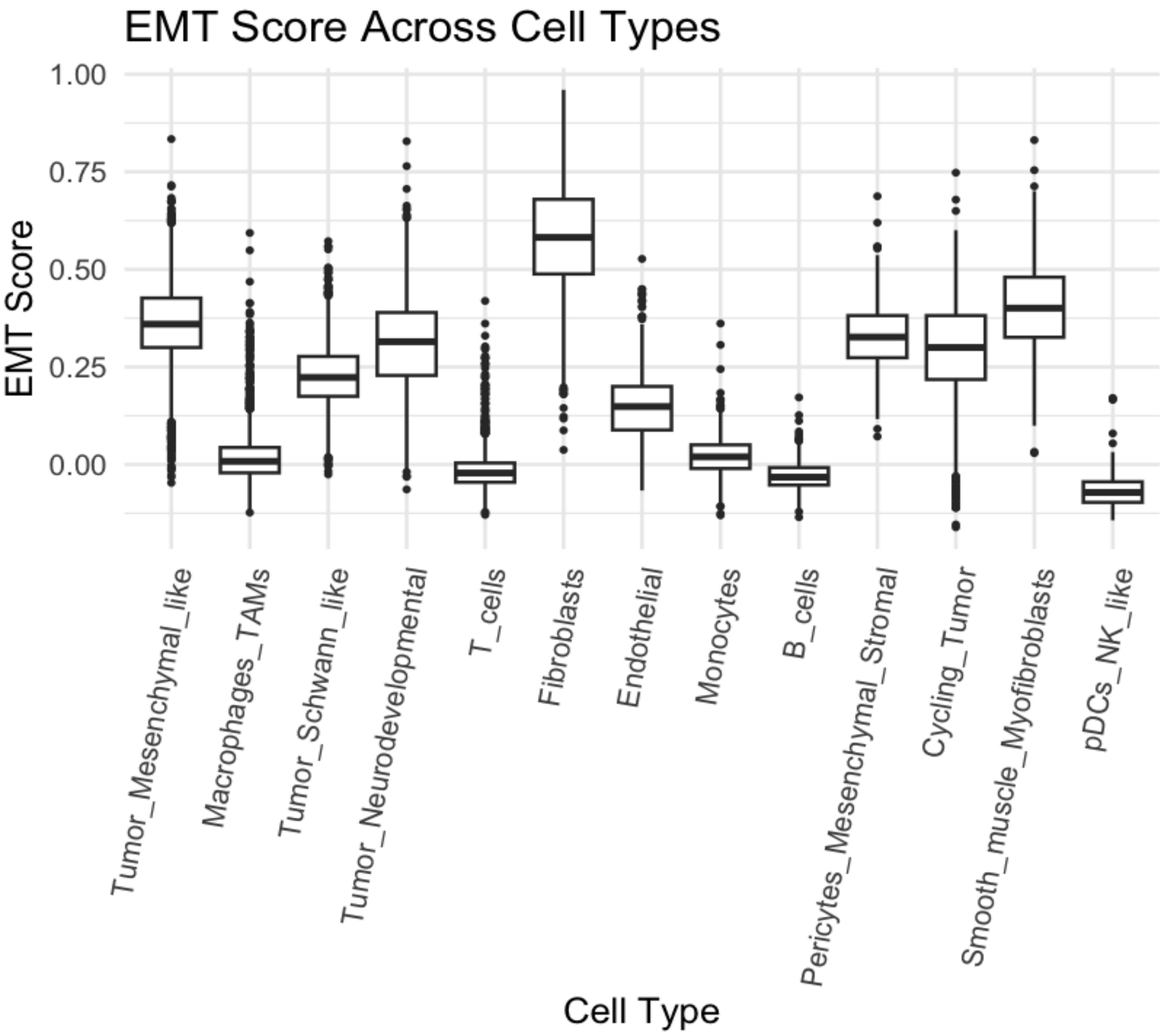

Supplementary Figure S10. EMT-like gene program across all cell clusters. Fibroblasts but also MPNST tumor cell clusters show higher EMT-like gene scores compared to other mesenchymal cells or immune cell subtypes, consistent with known biology.

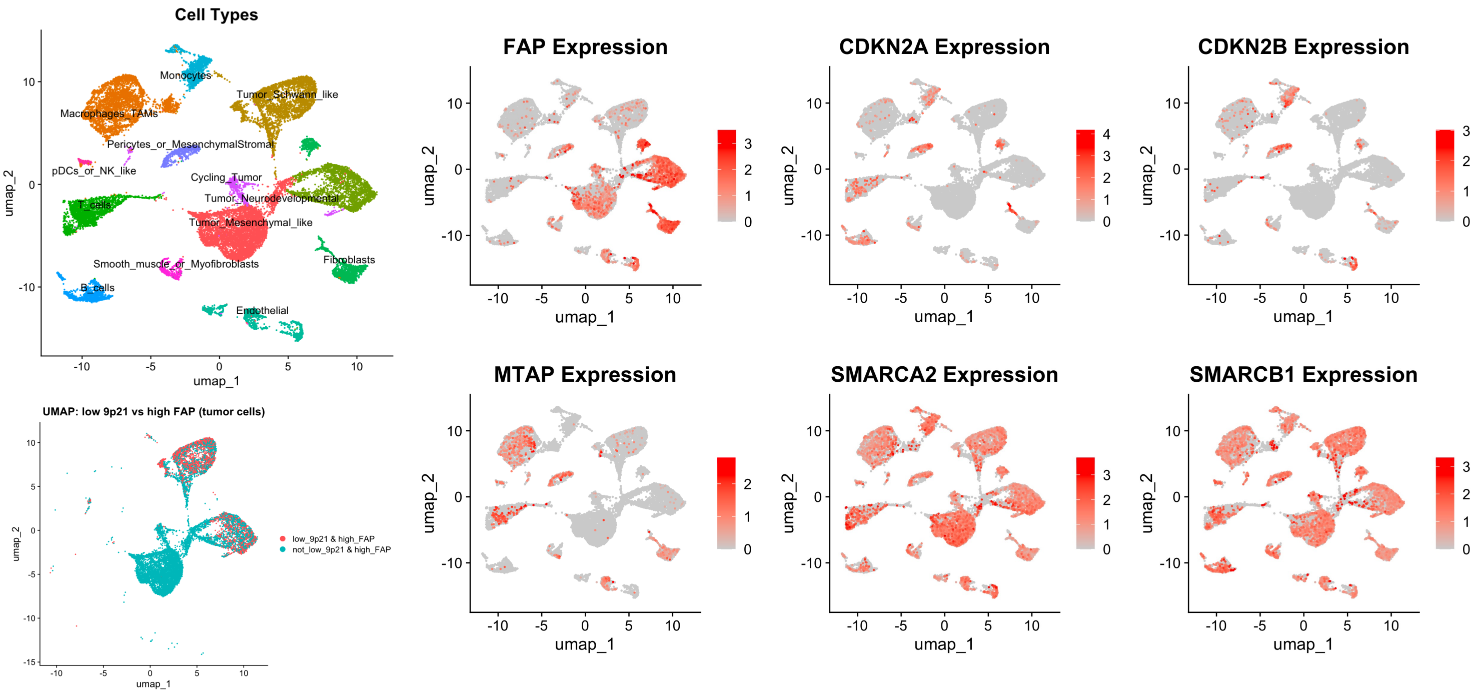

Supplementary Figure S11. Expression of 9p21 locus genes, *SMARCA2* and *SMARCB1* as well as *FAP* in publicly available single-cell RNA data. Here, we could identify tumor subpopulations with increased FAP and decreased 9p21 expression. However, it is important to notice that there are also some tumor cells with (partially) retained 9p21 expression and high FAP expression, suggesting that FAP upregulation is a ‘broader’ malignancy marker than CDKN2A/B etc. loss. However, sample size is here quite limited and further studies should evaluate this.

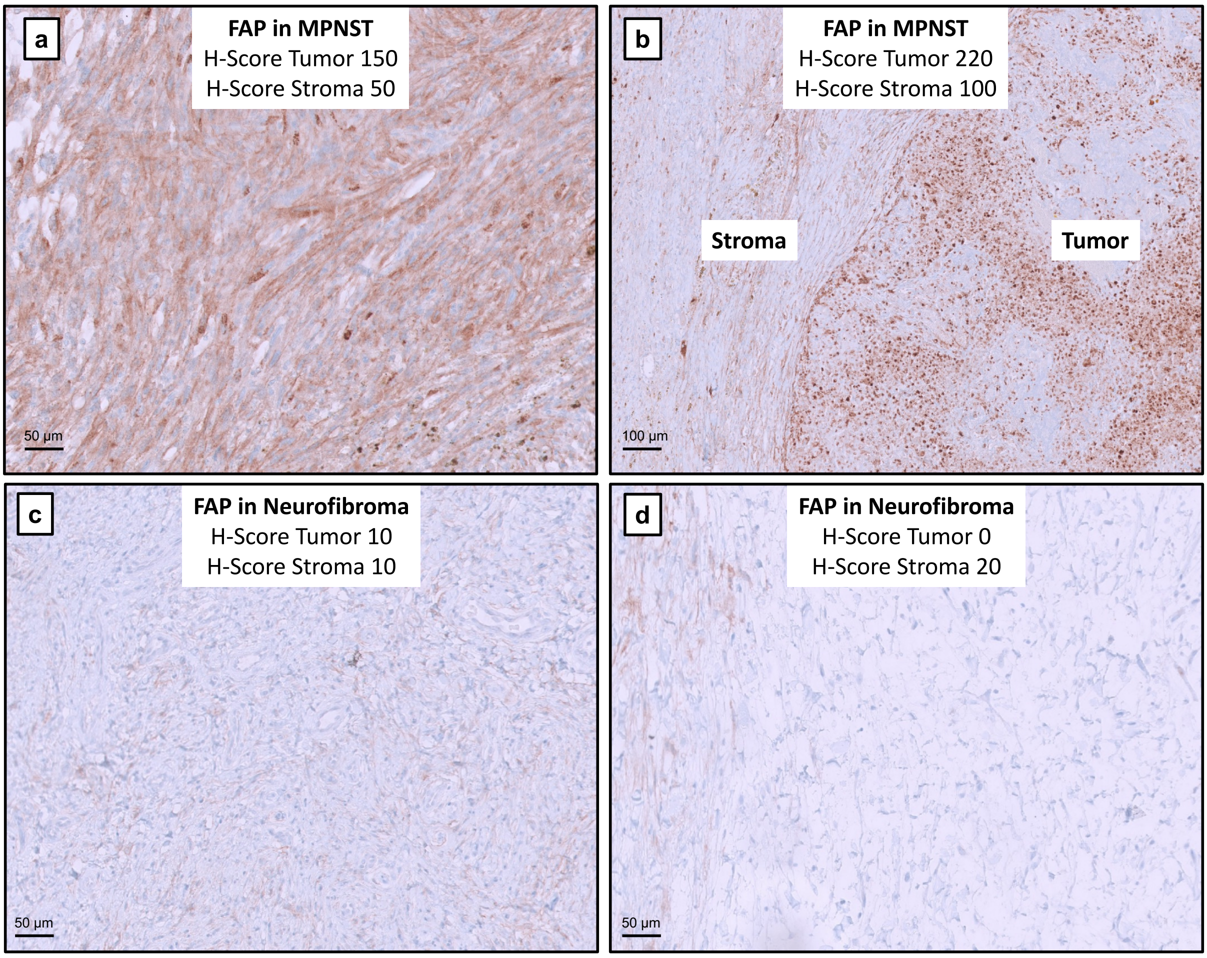

Supplementary Figure S12. Representative FAP immunohistochemistry for MPNSTs (a, b) and neurofibromas (c, d). Whereas MPNSTs exhibit strong membranous and cytoplasmic FAP expression on tumor cells as well as on cancer-associated fibroblasts in the adjacent stroma **(a, b)**, neurofibromas show absent to low FAP protein expression **(c, d)**. In **d**, perilesional tissue seems to exhibit faint FAP expression.

We used the well-established H-score for quantification of FAP immunohistochemistry: 1 × percentage of weak staining) + (2 × percentage of moderate staining) + (3 × percentage of strong staining). The H-score ranges from 0 to 300.

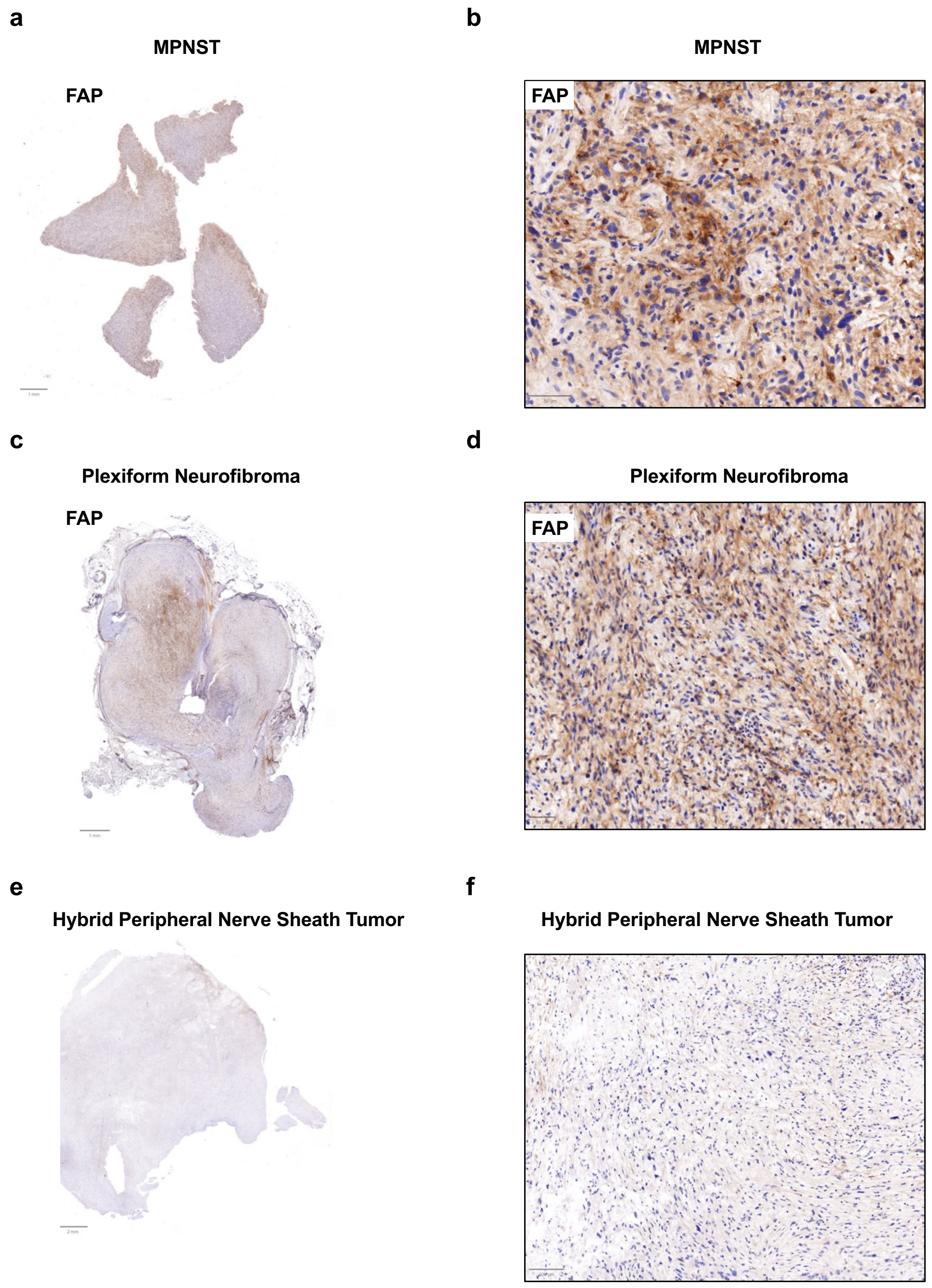

Supplementary Figure S13. FAP expression across different peripheral nerve sheath tumors: a MPNST, **b** plexiform neurofibroma, **c** hypbrid peripheral nerve sheath tumor (HPNST)

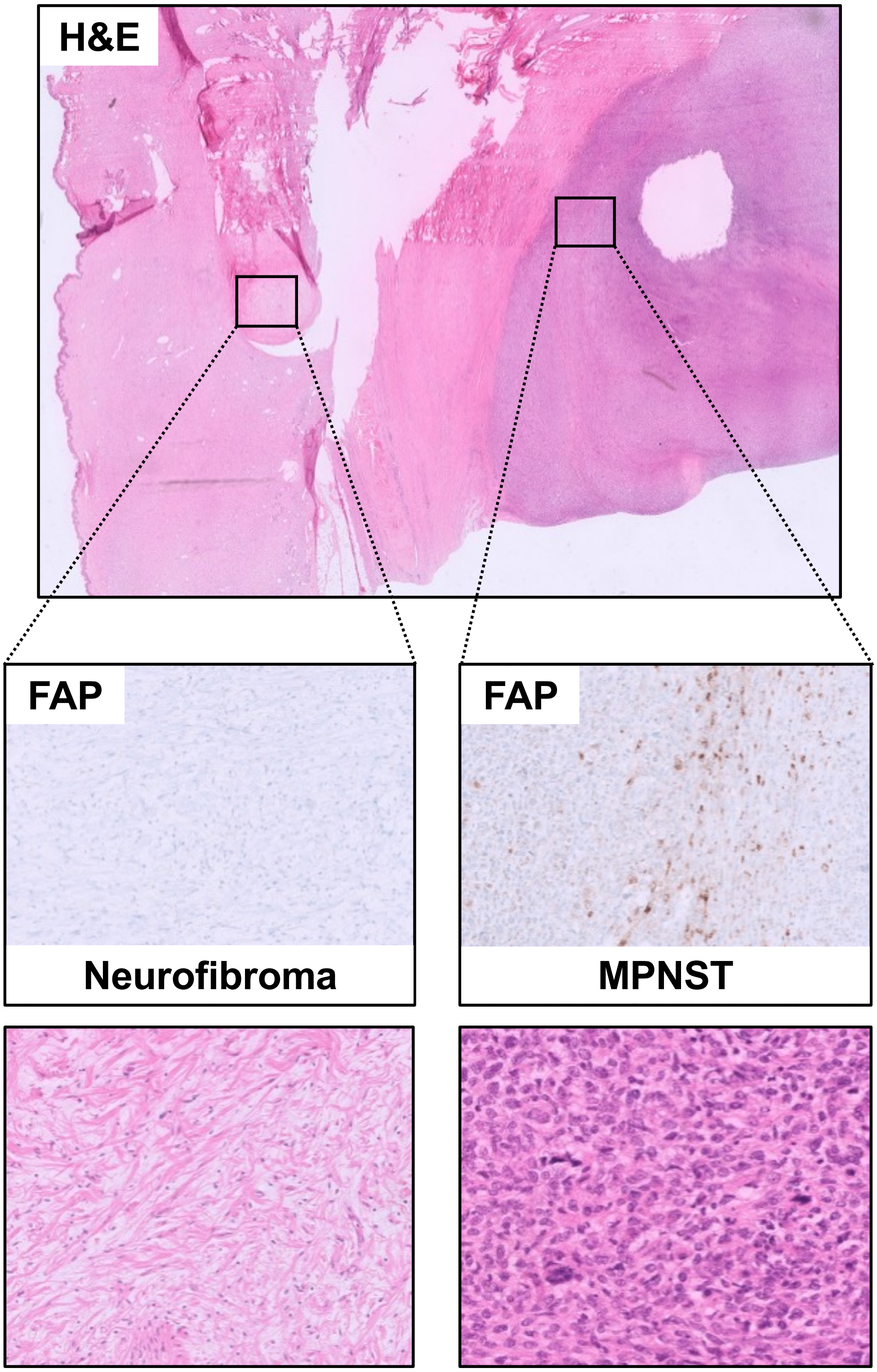

Supplementary Figure S14. Neurofibroma adjacent to MPNST in NF1 patient. Whereas the benign neurofibroma shows no FAP expression, MPNST tumor cells show heterogenous but partially strong FAP expression.

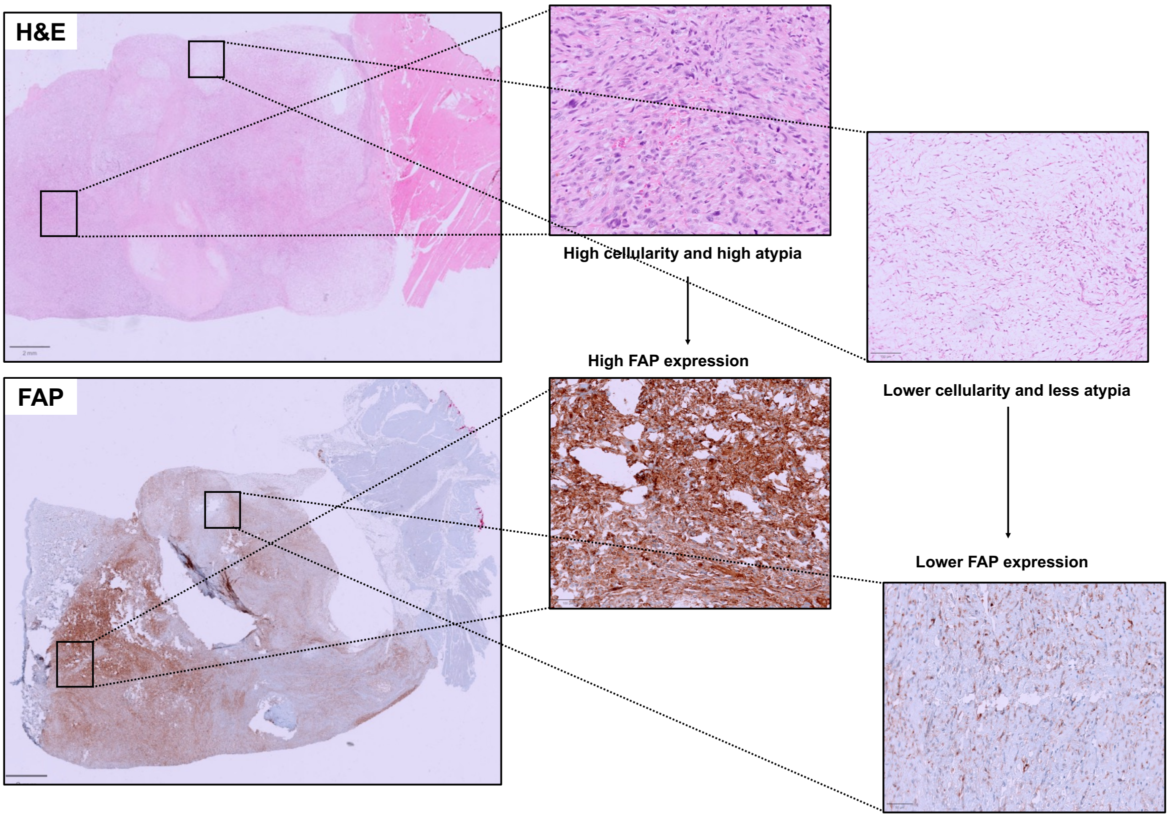

Supplementary Figure S15. Better differentiated areas within a MPNST case exhibit lower FAP expression. In this NF1-associated MPNST case, areas with high cellularity and high atypia display high tumor-cell-intrinsic FAP expression, whereas better differentiated areas (potentially remnants of pre-existing neurofibromas) show lower FAP expression.

FAP Immunohistochemistry

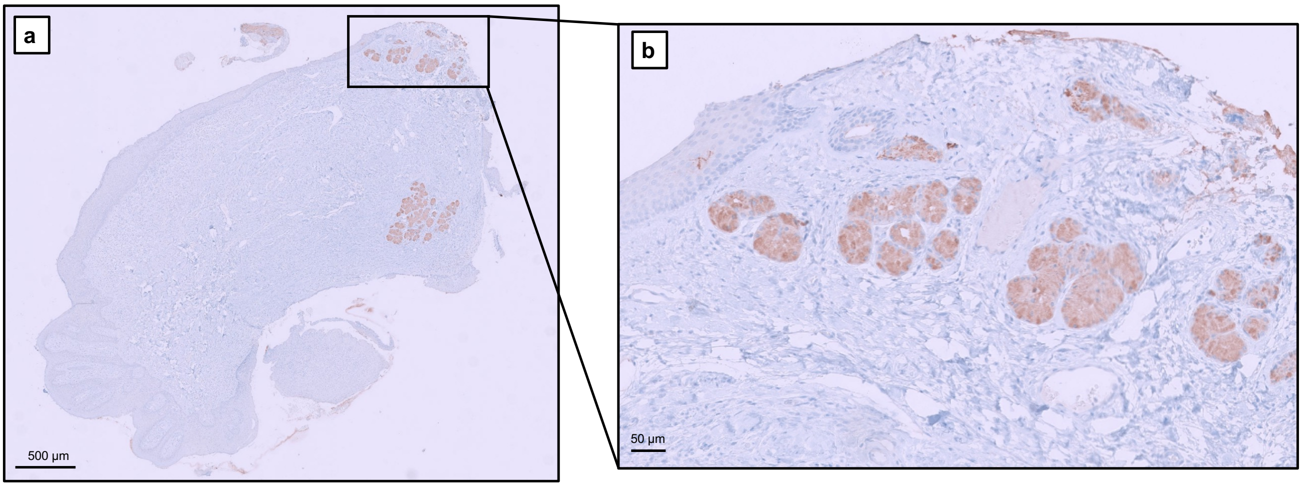

c

Summary of BLAST alignment between human FAP and DPP4:
51.9% sequence identity over 98% query coverage (E-value: 0.0), indicating high structural homology

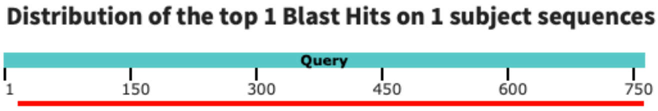

Supplementary Figure S16. FAP immunoreactivity and sequence similarity with DPP4. **a** Overview image of a submucosal neurofibroma covered by non-keratinized stratified squamous epithelium. Notably, there is no detectable FAP immunoreactivity in the neurofibroma. However, eccrine sweat glands at the periphery show distinct FAP signal. **b** Higher magnification of eccrine glands demonstrating localized FAP immunoreactivity. **c** BLAST alignment summary (https://blast.ncbi.nlm.nih.gov/Blast.cgi) and distribution plot comparing human FAP and DPP4. The alignment shows 51.9% sequence identity across 98% of the sequence (E-value: 0.0), supporting potential cross-reactivity due to structural homology.

UniProt DPP4_Human: https://www.uniprot.org/uniprotkb/P27487/entry

UniProt FAP_Human: https://www.uniprot.org/uniprotkb/Q12884/entry

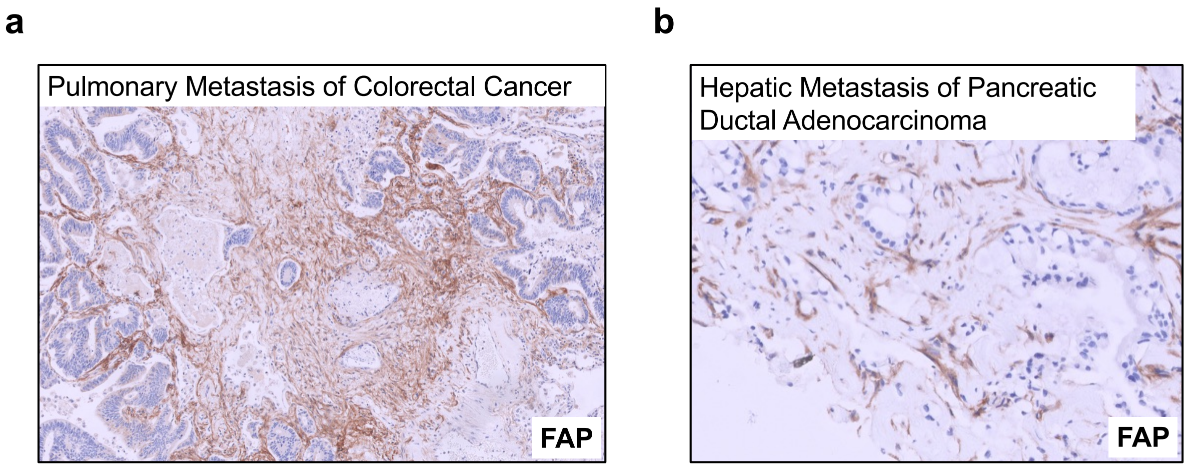

Supplementary Figure S17. FAP expression in cancer-associated fibroblasts in carcinomas. a Pulmonary metastasis of colorectal cancer with no FAP expression in tumor glands but high FAP expression in cancer-associated fibroblasts, **b** Hepatic metastasis of pancratic ductal adenocarcinoma with now FAP expression in tumor cells but strong and distinct FAP expression in surrounding fibroblasts.

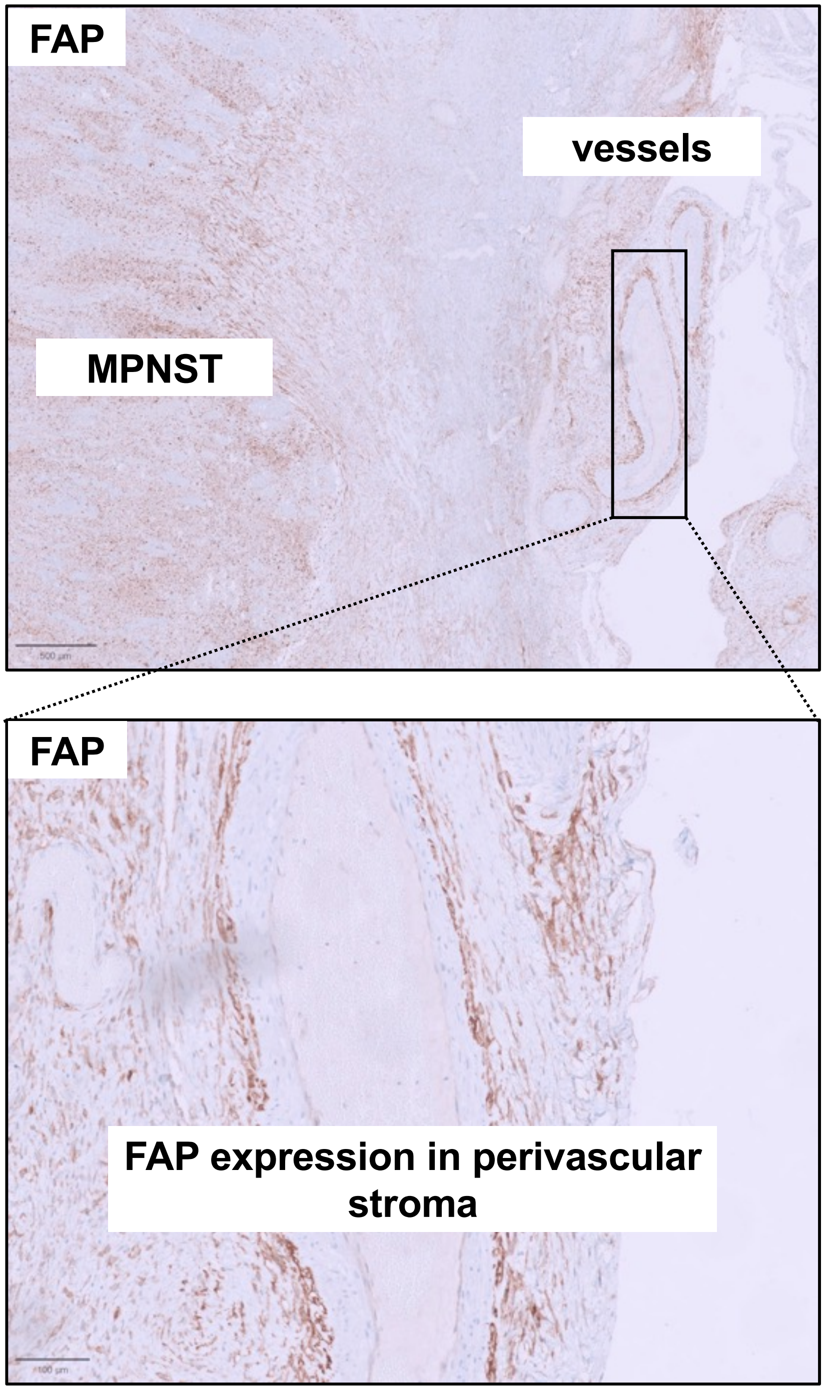

Supplementary Figure 18. FAP expression in MPNST tumor cells and perivascular stroma cells. FAP is strongly expressed in MPNST tumor cells but also in perivascular stromal cells.

Supplementary Table S1. Pairwise Comparisons of Sarcoma Types in TCGA Regarding FAP mRNA Expression

Comparison	Z	P.unadj	P.adj
DDLPS - Desmoid	-2,2106193	0,02706221	0,568306459
DDLPS - LMS	2,03877887	0,0414721	0,870914143
Desmoid - LMS	2,69805744	0,00697454	0,146465353
DDLPS - MFS	-1,4715862	0,14113266	1
Desmoid - MFS	1,68441438	0,09210161	1
LMS - MFS	-3,0790986	0,00207628	0,043601873
DDLPS - MPNST	1,32819872	0,18411247	1
Desmoid - MPNST	2,64246626	0,00823047	0,172839797
LMS - MPNST	0,39844772	0,69030019	1
MFS - MPNST	2,12981737	0,03318669	0,696920581
DDLPS - SS	4,51992356	6,1862E-06	0,00012991
Desmoid - SS	4,0505105	5,1106E-05	0,001073226
LMS - SS	3,64818874	0,0002641	0,005546008
MFS - SS	5,07722922	3,8298E-07	8,04255E-06
MPNST - SS	2,33269861	0,01966397	0,412943352
DDLPS - UPS/MFH	-1,3429181	0,1792985	1
Desmoid - UPS/MFH	1,84536212	0,06498488	1
LMS - UPS/MFH	-3,4368411	0,00058854	0,012359355
MFS - UPS/MFH	0,37935586	0,70442363	1
MPNST - UPS/MFH	-2,0298708	0,04236968	0,889763257
SS - UPS/MFH	-5,2157892	1,8304E-07	3,84376E-06

**Supplementary Table S2. Pairwise Comparisons of Immunhistochemical Analyses.**			
**Region**	**Comparison**	**p**	**q**
Stroma	Neurofibroma vs MPNST	0.000035	0.000211
Stroma	Neurofibroma vs Plexiform neurofibroma	0.001000	0.003000
Stroma	Neurofibroma vs HPNST	0.030000	0.059000
Stroma	Plexiform neurofibroma vs MPNST	0.201000	0.302000
Stroma	HPNST vs MPNST	0.407000	0.488000
Stroma	Plexiform neurofibroma vs HPNST	1.000000	1.000000
Tumor	Neurofibroma vs MPNST	0.000018	0.000109
Tumor	Neurofibroma vs Plexiform neurofibroma	0.000314	0.000942
Tumor	Plexiform neurofibroma vs MPNST	0.015000	0.029000
Tumor	Neurofibroma vs HPNST	0.029000	0.043000
Tumor	HPNST vs MPNST	0.036000	0.043000
Tumor	Plexiform neurofibroma vs HPNST	0.092000	0.092000
